# Supplementary material for: NAP1 switches from an activator to a limiter of interferon induction by trapping TBK1 in condensates
Source: Nat Commun. 2026 Aug 19;17:9915. doi: 10.1038/s41467-026-76769-w (PMC13582825; doi:10.1038/s41467-026-76769-w)
Supplement: Supplementary file 1 [file 41467_2026_76769_MOESM1_ESM.pdf]

Supplementary Information

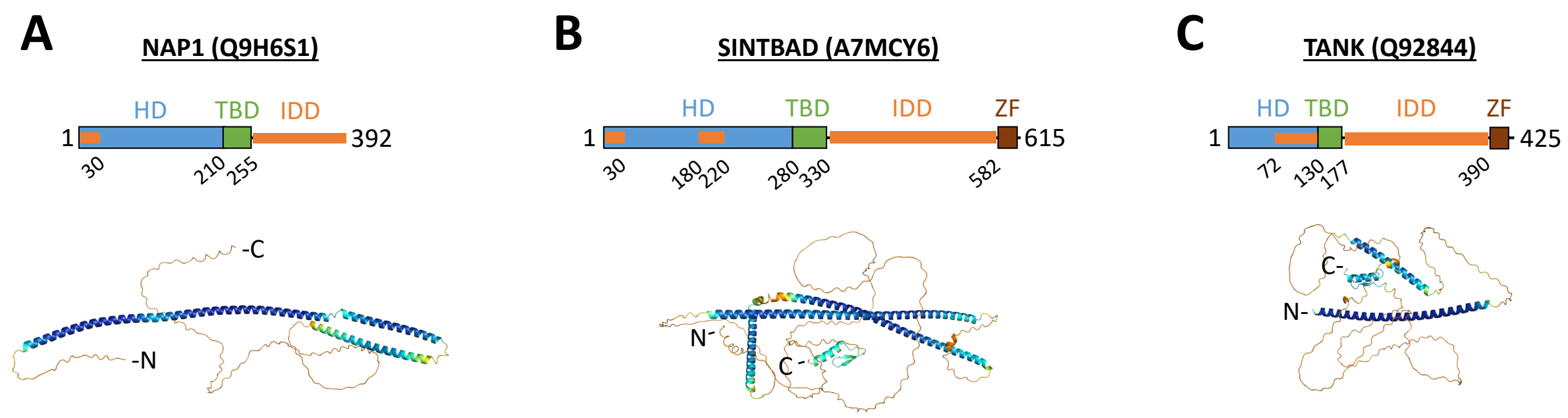

Supplementary Fig. 1 | Modular organization of NAP1, SINTBAD and TANK
A-C) Bar diagram showing the domain organization of NAP1 (**A**), SINTBAD (**B**) and TANK (**C**). HD, homodimerization domain; IDD, intrinsically disordered domain; TBD, TBK1 binding domain; ZF, zinc finger. The Alphafold3 prediction of the structure of each protein is also presented. Putative intrinsically disordered regions were predicted based on Alphafold3 structures using the MobiDB software[1].

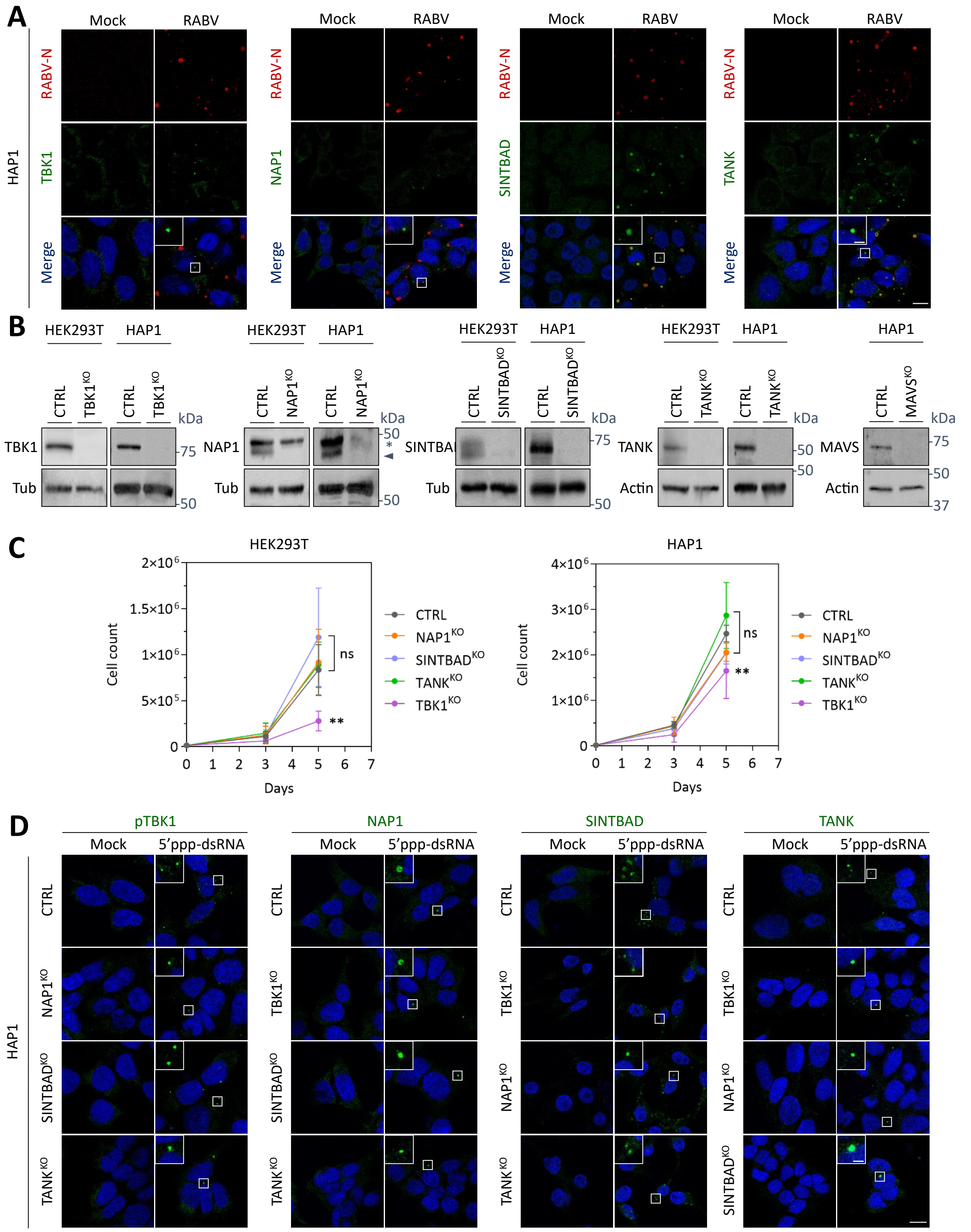

Supplementary Fig. 2 | Detection of danger signals induces condensation of TBK1, NAP1, SINTBAD and TANK

A) HAP1 cells were infected with RABV (MOI=3) or not (Mock). At 16 h p.i., cells were fixed before co-staining with anti-N and either anti-pTBK1, anti-NAP1, anti-SINTBAD or anti-TANK antibodies. Representative confocal images (n=3).

B) Parental (CTRL) and indicated KO HEK293T or HAP1 cells were lysed. Extracts were analyzed by WB with either anti-TBK1, anti-NAP1, anti-SINTBAD, anti-TANK or anti-MAVS antibody. Anti-Tubulin or anti-Actin antibodies were used as loading control. For the anti-NAP1 antibody, the star indicates

an unspecific band whereas the arrow indicates the band corresponding to NAP1.

C) The growth curves of parental (CTRL) and KO HEK293T and HAP1 cells ($TBK1^{KO}$, $NAP1^{KO}$, $SINTBAD^{KO}$ and $TANK^{KO}$) were drawn by counting cells after 3 and 5 days of culture in 3 independent experiments. Statistical differences with CTRL cells are shown. ns = non-significant; **P < 0.01 (two-way ANOVA followed by Tukey's multiple comparisons test (two-sided)).

D) Parental (CTRL) and KO HAP1 cells ($TBK1^{KO}$, $NAP1^{KO}$, $SINTBAD^{KO}$ and $TANK^{KO}$) were incubated with medium containing Lipofectamine 3000 alone (Mock) or transfected with 5'ppp-dsRNA using Lipofectamine 3000 (5'ppp-dsRNA). After 6 h, cells were fixed before staining with anti-pTBK1, anti-NAP1, anti-SINTBAD or anti-TANK antibody and analyzed by confocal microscopy.THP-1 cells (differentiated with PMA for 48 h) were treated with diABZI for 6 h. Cells were fixed before staining with anti-pTBK1 (S172), anti-NAP1, anti-SINTBAD or anti-TANK antibodies. Representative confocal images (n=3).

On microscopy images, square boxes have been magnified (3X) at the top left. Scale bars: 10µm and 2 µm for insets. Source data are provided as a Source Data file.

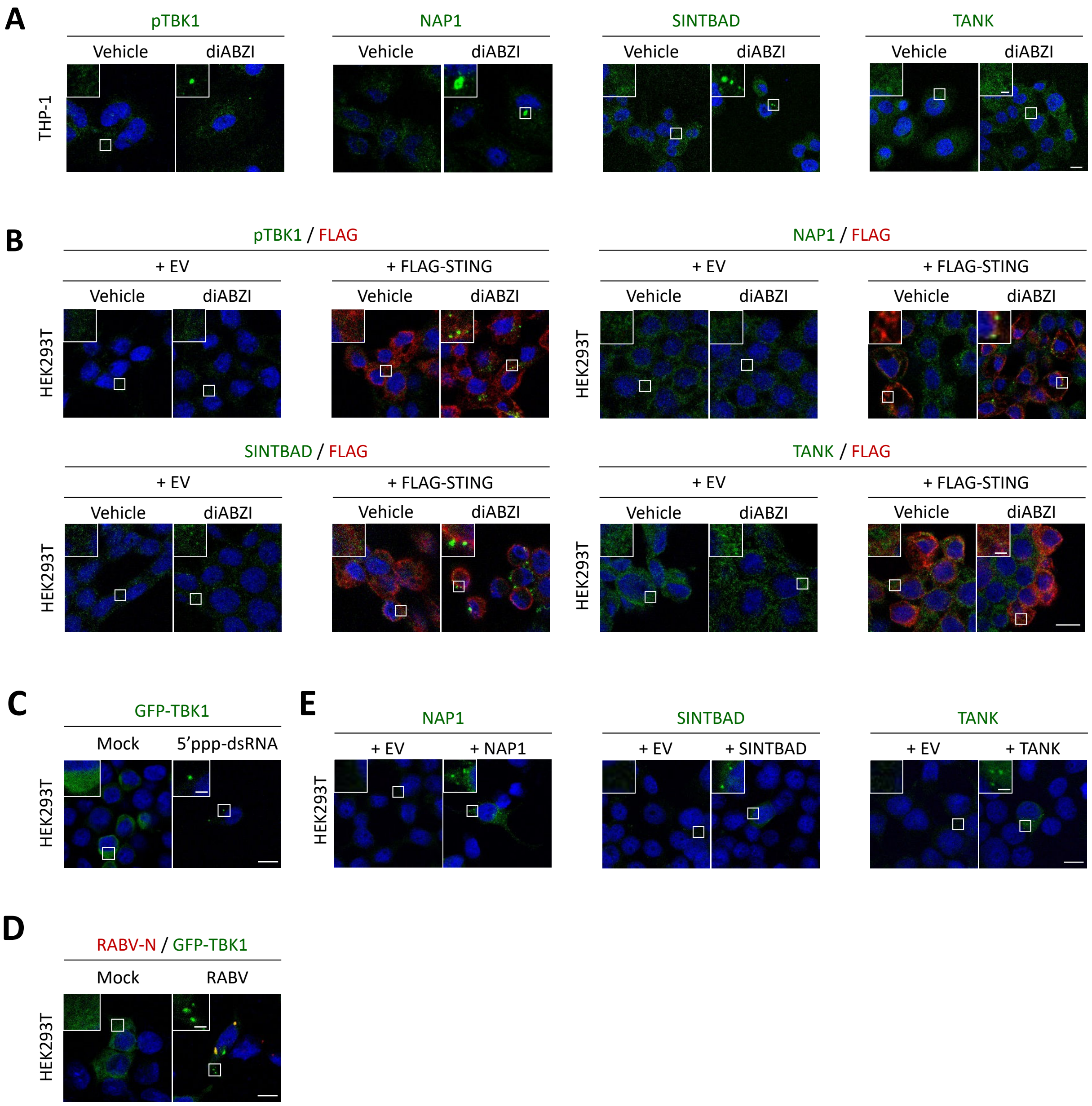

Supplementary Fig. 3 | Detection of danger signals as well as ectopic expression induce condensation of TBK1, NAP1, SINTBAD and TANK

A) THP-1 cells (differentiated with PMA for 48 h) were treated with diABZI for 6 h. Cells were fixed before staining with anti-pTBK1 (S172), anti-NAP1, anti-SINTBAD or anti-TANK antibodies. Representative confocal images (n=3).

B) HEK293T cells were transfected with a plasmid encoding FLAG-STING or an empty vector (EV). After 24 h, cells were treated with diabZI for 6h. Cells were fixed before staining with anti-FLAG and either anti-pTBK1 (S172), anti-NAP1, anti-SINTBAD or anti-TANK antibody. Representative confocal images (n=3).

C) HEK293T cells were transfected with plasmids encoding GFP-TBK1. After 18 h, cells were incubated with a medium containing Lipofectamine 3000 alone (Mock) or transfected with 5'ppp-dsRNA using Lipofectamine 3000 (5'ppp-dsRNA). After an additional 6h, cells were fixed. Representative confocal images (n=3).

D) HEK293T cells were infected (MOI=3) with RABV or not (Mock) and transfected with a plasmid encoding GFP-TBK1. At 16 h p.i., cells were fixed before staining with anti-N antibody. Representative confocal images (n=3).

E) HEK293T cells were transfected with plasmids encoding untagged versions of NAP1, SINTBAD or TANK (or EV). After 24 h, cells were fixed before staining with anti-NAP1, anti-SINTBAD or anti-TANK antibody. Representative confocal images (n=3).
On microscopy images, square boxes have been magnified (3X) at the top left. Scale bars: 10µm and 2 µm for insets.

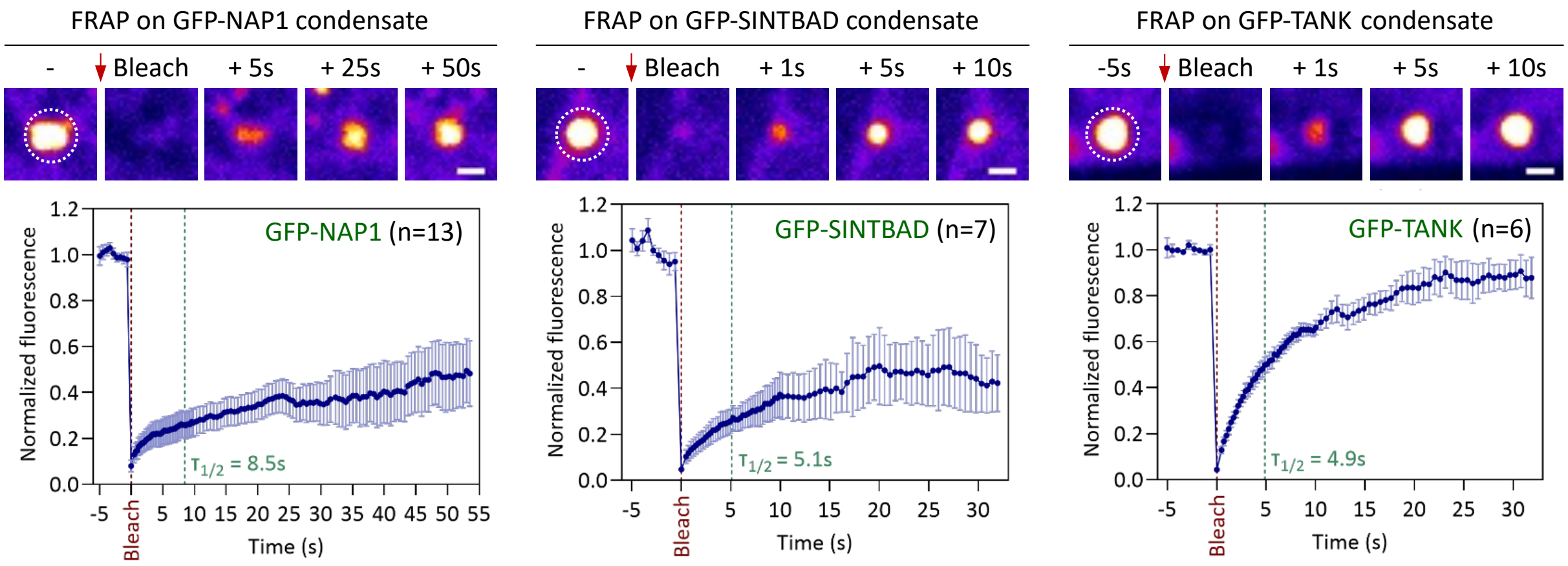

Supplementary Fig. 4 | Characterization of TBK1-adaptor protein condensates
HEK293T cells were transfected with plasmids encoding GFP-NAP1, GFP-SINTBAD or GFP-TANK. After 20 h, whole condensates were photobleached and the recovery of fluorescence was followed by a confocal spinning-disk microscope. A representative FRAP experiment is shown for each protein and the photobleached region (Ø = 3 µm) is indicated. For each protein, the mean curve and the SD of the indicated number of FRAP experiments (from 3 independent experiments) is shown. Scale bars: 2µm. Source data are provided as a Source Data file.

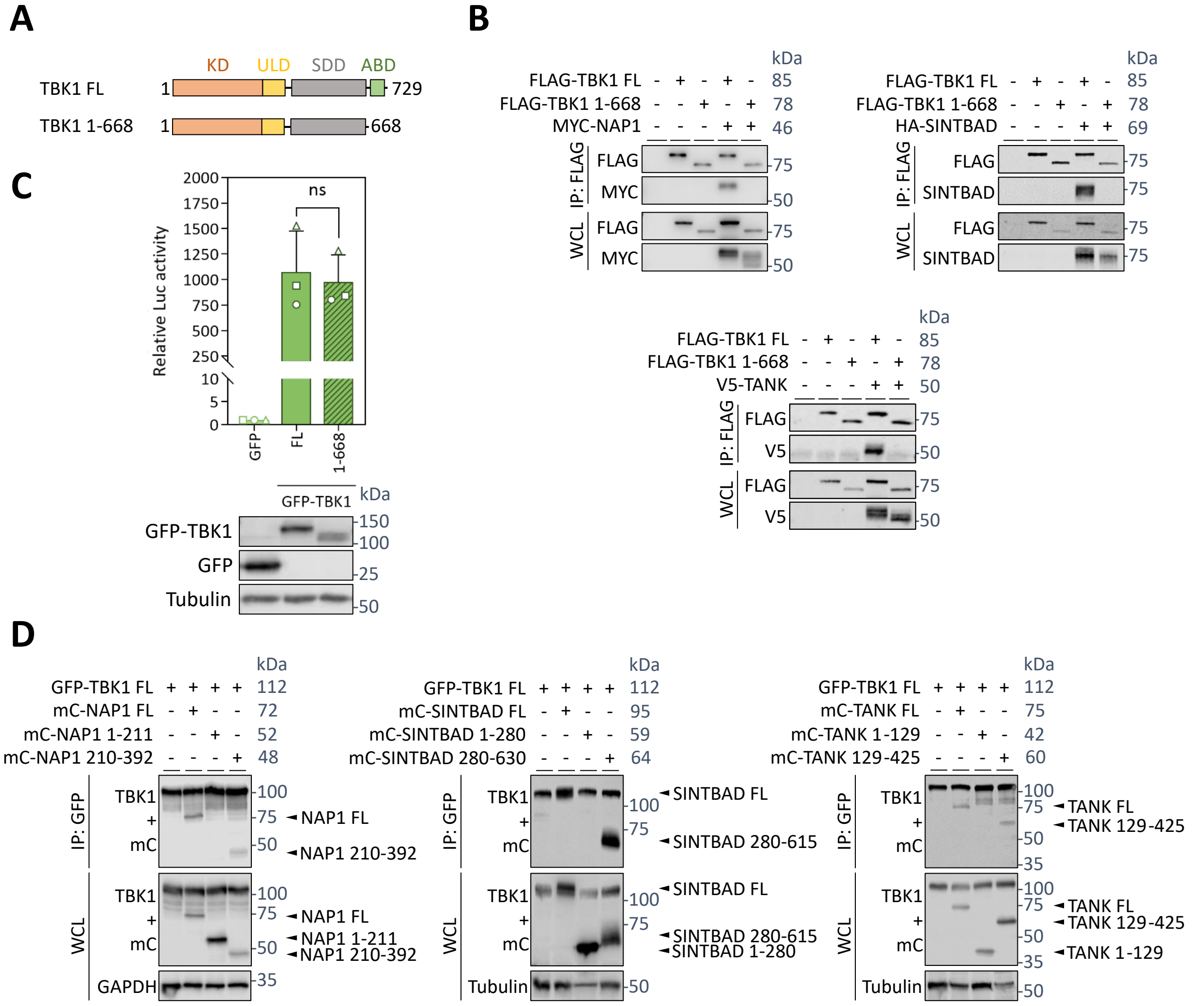

Supplementary Fig. 5 | Characterization of TBK1 and TBK1-adaptor protein truncations

A) Bar diagram showing the domain organization of TBK1. The ABD domain is absent in deletion mutant TBK1 1-668.

B) HEK293T cells were co-transfected with plasmids encoding FLAG-TBK1 (FL or 1-668) and MYC-NAP1 (upper left panel), HA-SINTBAD (upper right panel) or V5-TANK (bottom left panel) for 24 h. After immunoprecipitation of TBK1 (FL or 1-668) with an anti-FLAG antibody, cell lysates (WCL) and immunoprecipitated proteins (IP) were analyzed by WB with anti-FLAG and anti-MYC, anti-SINTBAD or anti-V5 antibodies.

C) HEK293T cells were transfected with plasmids encoding GFP-TBK1 (FL or 1-668) or GFP alone and the plasmids of the Luc reporter assay. After 24 h, the induction of the IFN pathway was assessed using the Luc reporter assay (activation of the IFN-β promoter). Cell lysates were analyzed by WB using anti-GFP and anti-Tubulin antibodies. The bars represent the mean ± SD normalized to the GFP condition of 3 independent experiments. ns =non-significant (Two-sided Student t-test).

D) HEK293T cells were co-transfected with plasmids encoding GFP-TBK1 and indicated truncations of mC-NAP1 (left panel), mC-SINTBAD (middle panel) or mC-TANK (right panel). After immunoprecipitation of TBK1 with an anti-GFP antibody, cell lysates (WCL) and immunoprecipitated proteins (IP) were analyzed by WB with anti-TBK1, anti-mC and anti-GAPDH or anti-Tubulin antibodies. It is worth noting that mC-SINTBAD FL and GFP-TBK1 FL co-migrate in SDS-PAGE despite different molecular weights.

Source data are provided as a Source Data file.

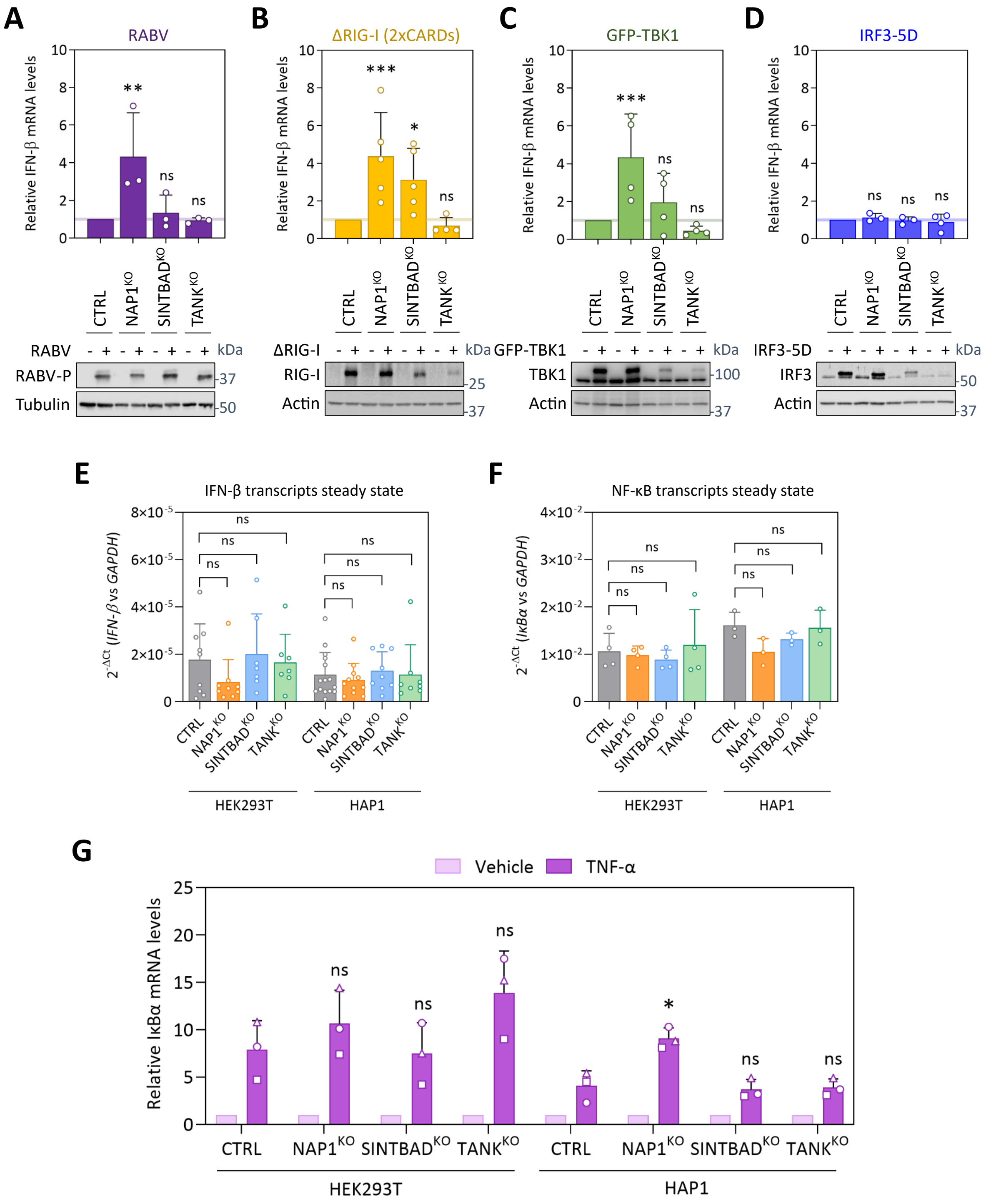

Supplementary Fig. 6 | Impact of TBK1-adaptor proteins deficiency on IFN-β and NF-κB pathway induction

A-D) Parental (CTRL) and KO HAP1 cells (NAP1^{KO}, SINTBAD^{KO}, and TANK^{KO}) were infected (MOI=3) with RABV (**A**), or transfected either with ΔRIG-I (**B**), GFP-TBK1 (**C**) or IRF3-5D (**D**) plasmids. At 16 h p.i. or 24 h p.t., cells were lysed and the relative levels of *IFN-β* mRNAs to *GAPDH* mRNAs were quantified by RT-qPCR. For each KO cell line, the *IFN-β* mRNA fold induction relative to the control cells is indicated. The bars represent the mean ± SD of 3 (**A** and **D**), 4 (**C**) or 5 (**B**) independent experiments and the corresponding data points are shown. Cell lysates were also analyzed by WB using anti-P, anti-RIG-I, anti-GFP, anti-IRF3, anti-Actin, or anti-Tubulin antibodies.

E) IFN-β transcripts steady state. Untreated parental (CTRL) and KO HEK293T and HAP1 cells (NAP1^{KO},

SINTBAD^{KO}, and TANK^{KO}) were lysed. The relative levels of *IFN-β* mRNAs to *GAPDH* mRNAs were quantified by RT-qPCR ($2^{-\Delta Ct}$ method). The bars represent the mean ± SD of 3 independent experiments and the corresponding data points are shown.

F) NF-κB transcripts steady state. Untreated parental (CTRL) and KO HEK293T and HAP1 cells (NAP1^{KO}, SINTBAD^{KO}, and TANK^{KO}) were lysed. The relative levels of *IκBα* mRNAs to *GAPDH* mRNAs were quantified by RT-qPCR ($2^{-\Delta Ct}$ method). The bars represent the mean ± SD of 3 independent experiments and the corresponding data points are shown.

G) Parental (CTRL) and KO HEK293T and HAP1 cells (NAP1^{KO}, SINTBAD^{KO}, and TANK^{KO}) were treated or not (vehicle) with TNF-α. After 1h, cells were lysed and the relative levels of *IκBα*mRNAs to *GAPDH* mRNAs were quantified by RT-qPCR. For each cell line, the *IκBα mRN*A fold induction relative to the vehicle condition is indicated. The bars represent the mean ± SD of 3 independent experiments and the corresponding data points are shown.

ns = non-significant; *P < 0.05; **P < 0.01, ***P < 0.001 (ordinary one-way ANOVA followed by Dunnett's multiple comparisons test (two-sided) test [**all panels**]). Source data are provided as a Source Data file.

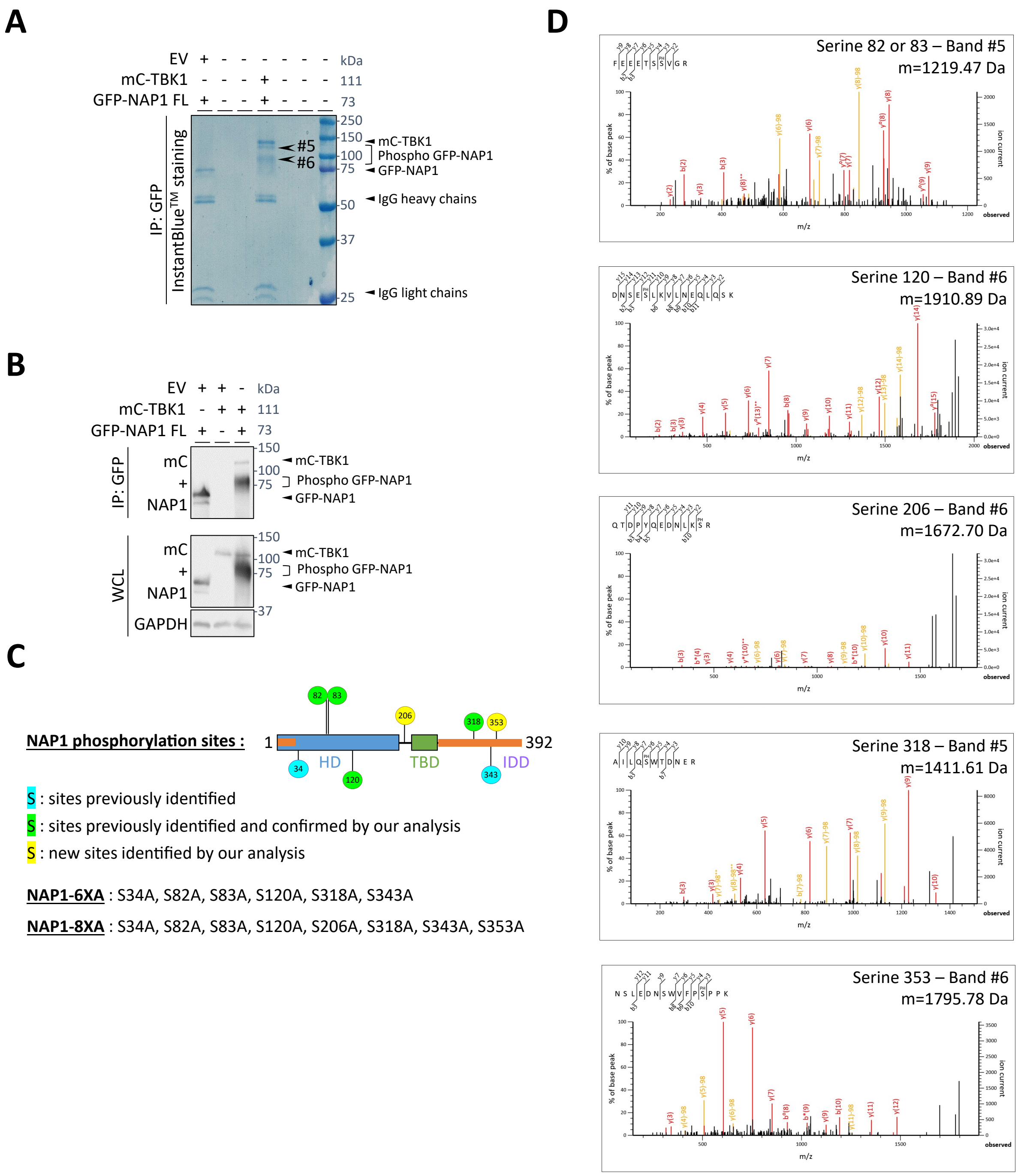

Supplementary Fig. 7 | Identification of NAP1 phosphorylation sites by LC-MS

A-B) HEK293T cells were co-transfected with plasmids encoding GFP-NAP1 and mC-TBK1 (or EV) for 24h. After cell lysis, GFP-NAP1 was immunoprecipitated using an anti-GFP antibody. **(A)** Immunoprecipitated proteins were separated by SDS-PAGE and stained by InstantBlue™ Coomassie. **(B)** Cell lysates (WCL) and immunoprecipitated proteins (IP) were also analyzed by WB with anti-NAP1, anti-mC and anti-GAPDH antibodies.

C) Scheme of NAP1 organization showing the position of phosphorylated serines identified in our analysis as either new phosphorylated sites (in yellow) or peptides previously identified in the literature (in green), or not identified in our analysis but previously reported (in blue). Each site has been mutated in alanine and two multiple mutants have been constructed (NAP1 6XA and NAP1 8XA) as indicated.

D) MS/MS spectra of phosphorylated peptides detected in digests of bands #5 and #6 of

phosphorylated GFP-NAP1 labeled on SDS-PAGE (**A**) identify phosphorylation sites S82 or S83, S120, S206, S318, and S353 on NAP1. The mass of the phosphorylated peptide is indicated. Ion peaks labeled with $^{++}$ have two positive charges, those labeled with ° are dehydrated peptides, those labeled with * are deaminated peptides. Source data are provided as a Source Data file and on PRIDE.

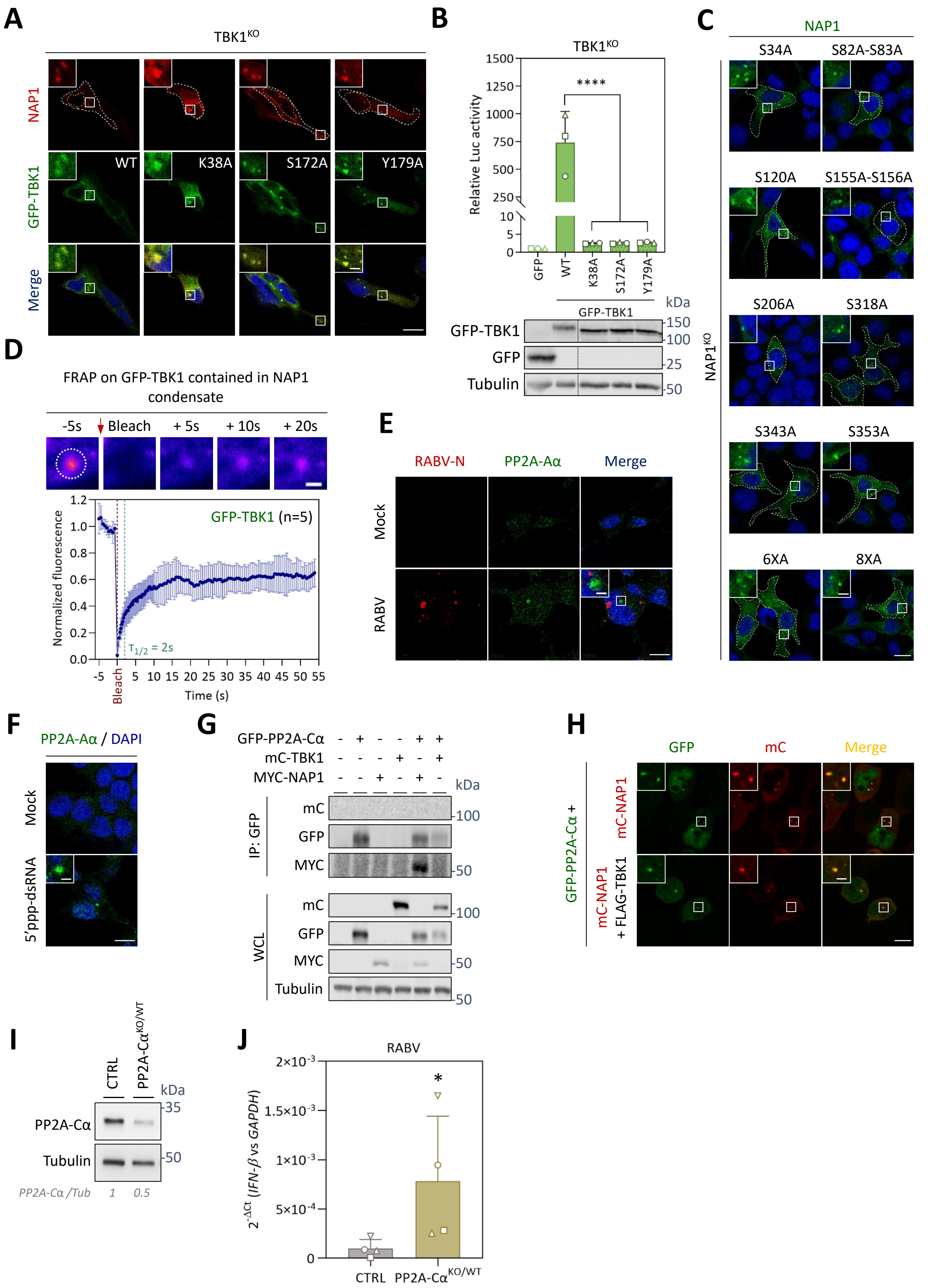

Supplementary Fig. 8 | Characterization of TBK1 and NAP1 mutants as well as the accumulation of PP2A phosphatase complex inside NAP1 condensates

A) TBK1^{KO} HEK293T cells were co-transfected with plasmids encoding NAP1 and GFP-TBK1 WT or mutants (K38A, S172A or Y179A). After 24 h, cells were fixed before staining with an anti-NAP1 antibody. Representative confocal images (n=3).

B) TBK1^{KO} HEK293T cells were transfected with plasmids encoding GFP-TBK1 WT or mutants (K38A, S172A or Y179A) or GFP alone, and the plasmids of the Luc reporter assay. After 24 h, the induction of the IFN pathway was assessed using the Luc reporter assay (activation of the IFN-β promoter). Cell lysates were analyzed by WB using anti-GFP and anti-Tubulin antibodies. The blot were spliced for presentation purpose and the splice sites are indicated by dotted lines. The bars represent the mean ± SD of 3 independent experiments and the corresponding data points are shown.
C) NAP1^{KO} HEK293T cells were transfected with plasmids encoding indicated NAP1 phosphorylation mutants. After 24 h, cells were fixed before staining with an anti-NAP1 antibody. Representative confocal images (n=3).
D) HEK293T cells were co-transfected with plasmids encoding GFP-TBK1 and mC-NAP1. After 20 h, whole condensates were photobleached and the recovery of fluorescence was followed. by a confocal spinning-disk microscope. A representative FRAP experiment is shown and the photobleached region (Ø=3 µm) is indicated. The mean curve and the SD of 5 FRAP experiments (from 3 independent experiments) is shown.
E) HEK293T cells were infected (MOI=3) with RABV or not (Mock). At 16 h p.i., cells were fixed before co-staining with anti-N and anti-PP2A-Aα antibodies. Representative confocal images (n=3).
F) HEK293T cells were incubated with a medium containing LyoVec alone (Mock) or transfected with 5'ppp-dsRNA using LyoVec (5'ppp-dsRNA). After 6 h, cells were fixed before staining with anti-PP2A-Aα antibody. Representative confocal images (n=3).
G) HEK293T cells were co-transfected with plasmids encoding GFP-PP2A-Cα (or EV) and mC-TBK1 or MYC-NAP1 for 24 h. After immunoprecipitation of PP2A-Cα with an anti-GFP antibody, cell lysates (WCL) and immunoprecipitated proteins (IP) were analyzed by WB with anti-mC, anti-GFP, anti-MYC and anti-Tubulin antibodies.
H) HEK293T cells were co-transfected for 20 h with plasmids encoding GFP-PP2A-Cα, mC-NAP1 and FLAG-TBK1 (or EV). Representative confocal images (n=3).
I) Parental (CTRL) and PP2A-Cα$^{KO/WT}$ HEK293T cells were lysed. Extracts were analyzed by WB with anti-PP2A-Cα and anti-Tubulin antibodies. The extinction level is indicated.
J) Parental (CTRL) and PP2A-Cα$^{KO/WT}$ HEK293T cells were infected (MOI=3) with RABV. At 16 h p.i., cells were lysed and the relative levels of IFN-β mRNAs to GAPDH mRNAs were quantified by RT-qPCR ($2^{-\Delta Ct}$ method). The bars represent the mean ± SD of 4 independent experiments and the corresponding data points are shown.
On microscopy images, square boxes have been magnified (3X) at the top left. Scale bars: 10µm and 2 µm for insets and FRAP experiments.
*P < 0.05; ***P < 0.001; (ordinary one-way ANOVA followed by Dunnett's multiple comparisons test (two-sided) [**B**] or Two-sided Mann-Whitney [**J**]). Source data are provided as a Source Data file.

A

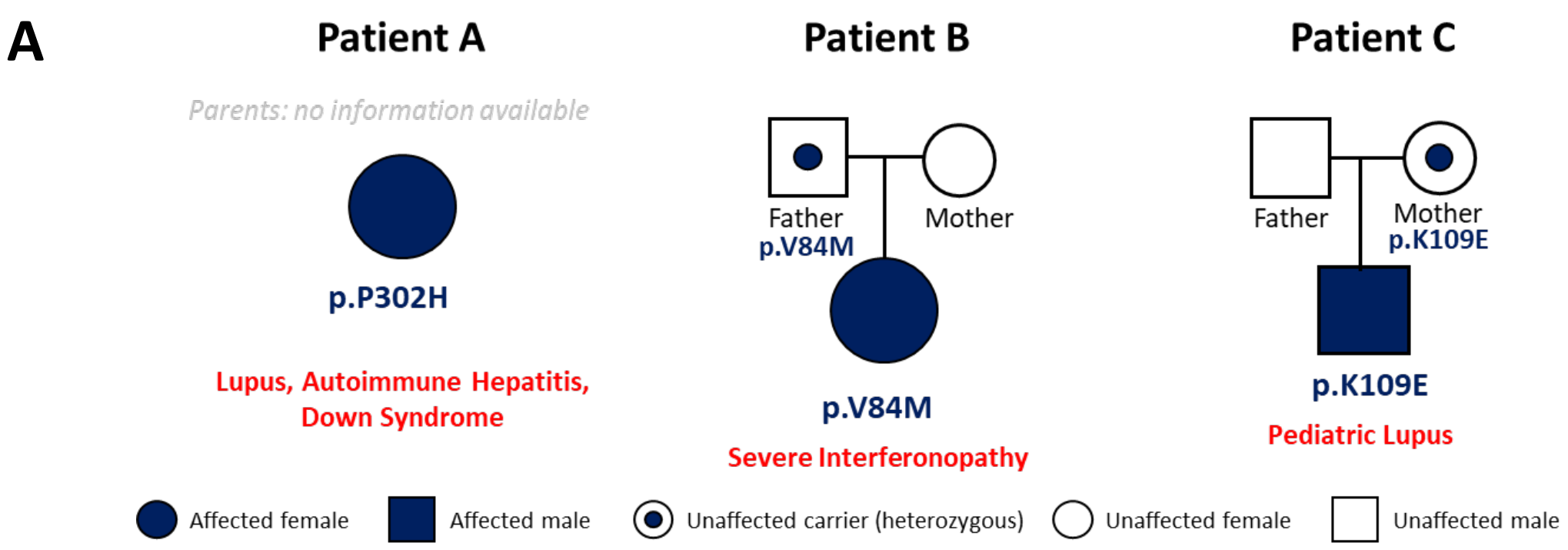

B

Patient	cDNA	Protein	CADD	PolyPhen	SIFT	MAF
A	c.250 G>A	p.V84M	24	0.175	0.11	$1.18x10^{-5}$
B	c.325 A>G	p.K109E	20	0.5	0.1	$6.36x10^{-7}$
C	c.905 C>A	p.P302H	24	0.945	0.01	0

C

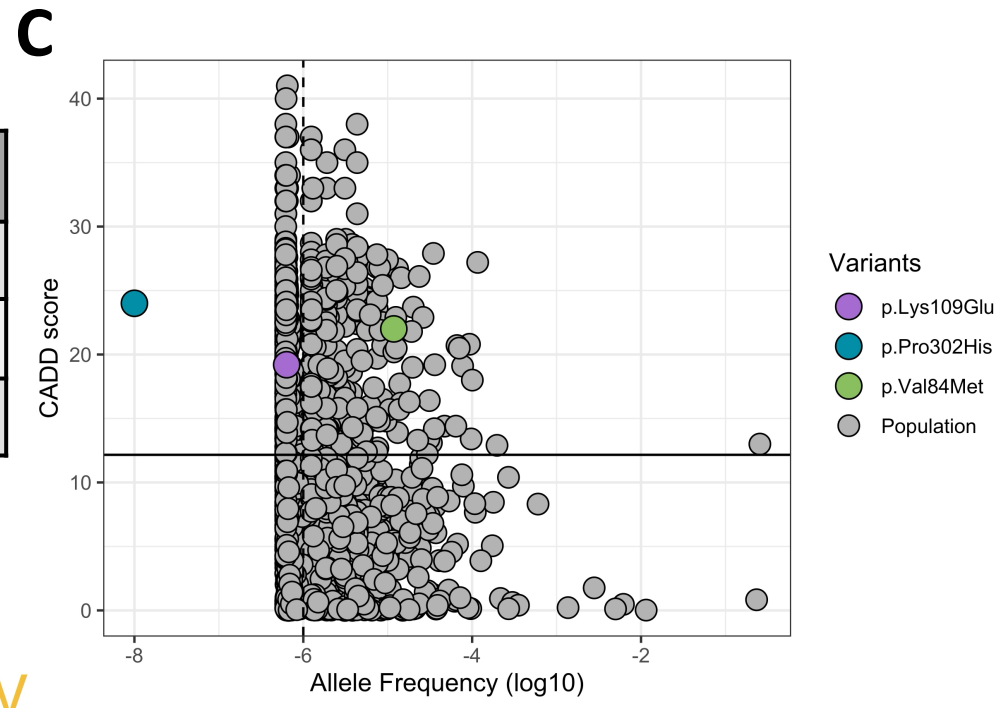

D

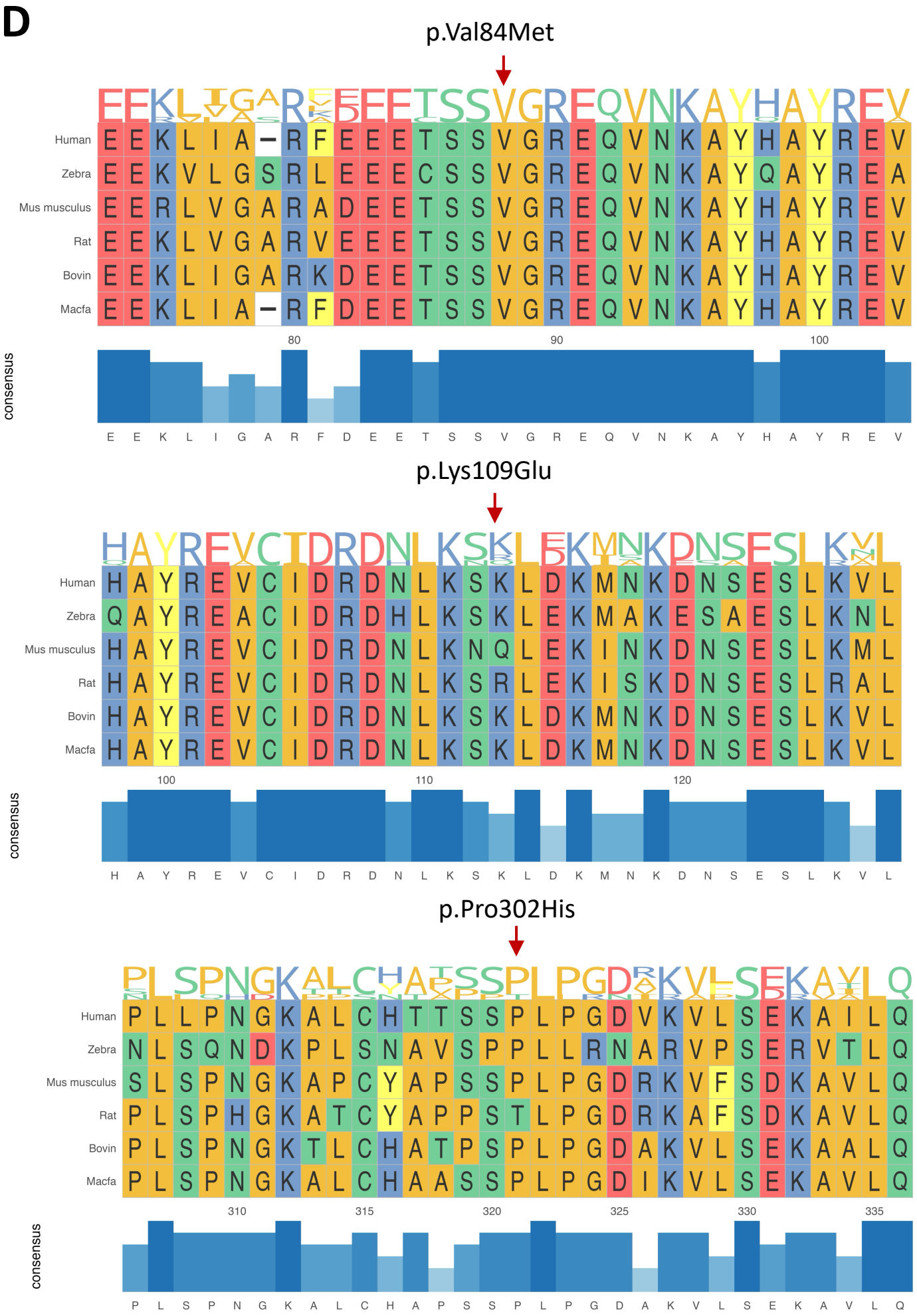

E

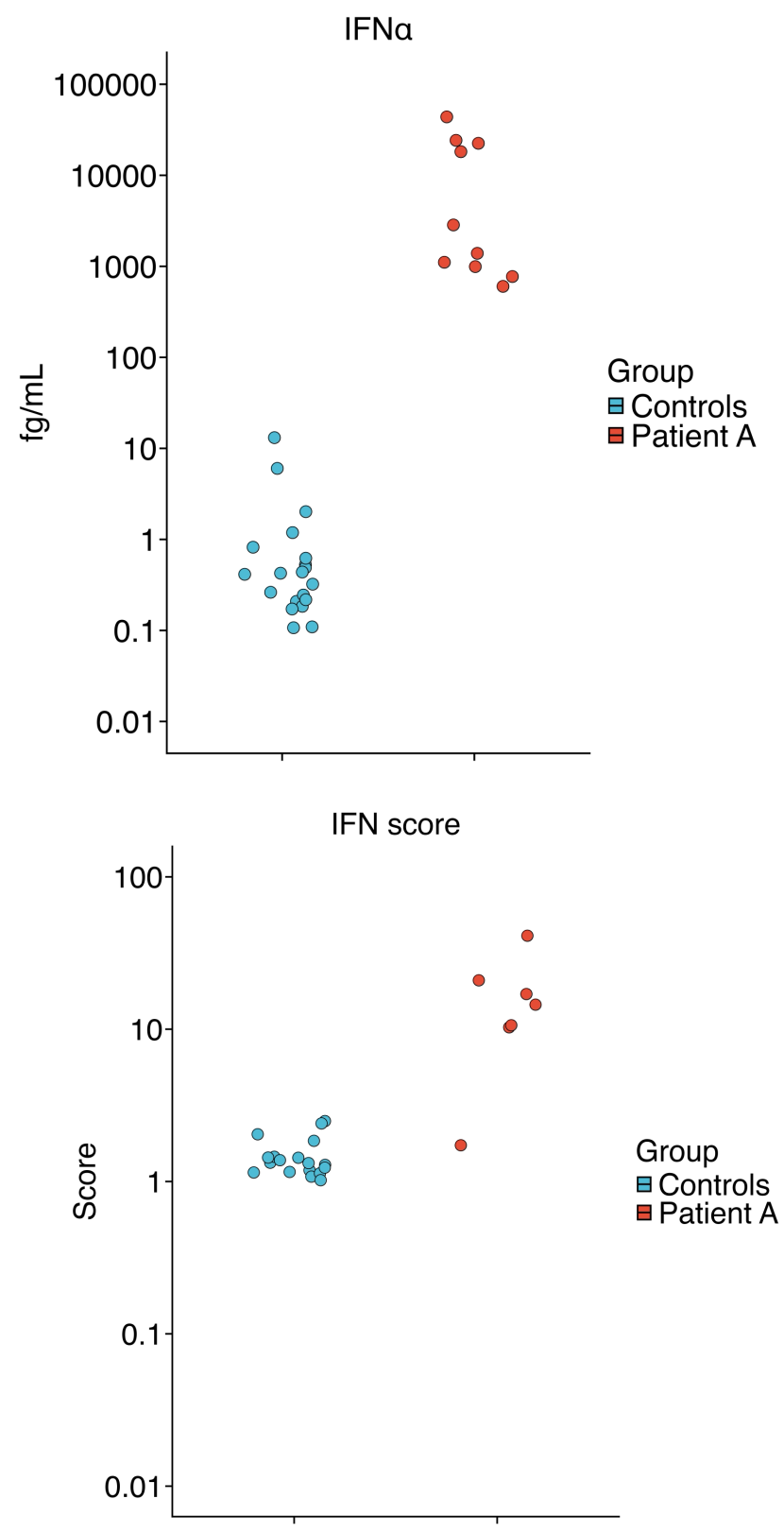

Supplementary Fig. 9 | Genotypic and phenotypic characterization of patients

A) Pedigrees of each patient of the study.

B) Table showing the characteristics of the different *AZI2*/NAP1 variants. The median allele frequency (MAF) in the general population based on gnomAD database (https://gnomad.broadinstitute.org/), the Combined Annotation Dependent Depletion score (CADD), the Sorting Intolerant from Intolerant score

(SIFT), and the PolyPhen score of the different variants are indicated.

C) CADD score versus Allele Frequency Dot Plot of each gnomAD v4.1 variants for AZI2. Patients' variants are highlited in colors. The horizontal bar represents the Mutation Significance Cutoff, a quantitative approach that provides gene-specific phenotypic impact cutoffs to improve the use of existing variant-level deleteriousness score. The vertical bar represents the cutoff of Allele Frequency 10^{-6}.

D) Conservation of amino-acid residues across different positions in NAP1 among mammalian species, the amino-acid substitued in each patients' variants is highlighted by the red arrow. The consensus score is a % of conservation across the depicted species. Macfa: *Macaca Fascicularis*.

E) SIMOA expression of IFNα in different plasma samples from the patient A and controls (upper panel). ISGs score by NanoString technology of different all blood longitudinal samples from patient A and controls (lower panel).

Source data are provided as a Source Data file.

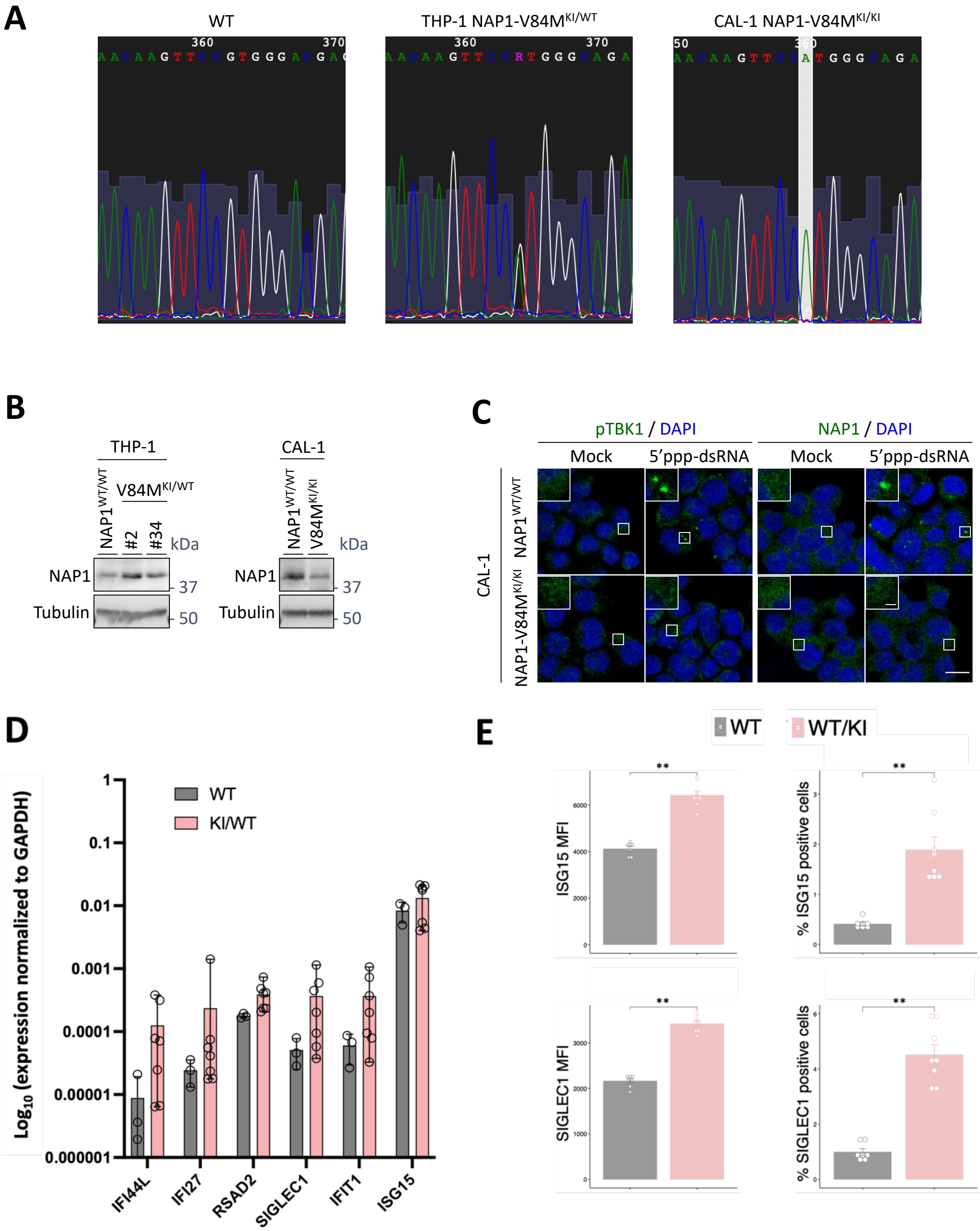

Supplementary Fig. 10 | NAP1 V84M variant does not form condensates in CAL-1 cells exposed to a danger signal and also induces spontaneous elevation of ISGs at the mRNA and protein level in THP-1 cells

A) Genome sequence electropherograms of THP-1 NAP1$^{WT/WT}$, THP-1 NAP1 V84M$^{KI/WT}$, and CAL-1 NAP1 V84M$^{KI/KI}$ cells showing the heterozygous status of THP-1 NAP1 V84M$^{KI/WT}$ and the homozygous status of CAL-1 NAP1 V84M$^{KI/KI}$ cells.

B) THP-1 NAP1$^{WT/WT}$, THP-1 NAP1 V84M$^{KI/WT}$, CAL-1 NAP1$^{WT/WT}$, and CAL-1 NAP1 V84M$^{KI/KI}$ cells were lysed. Extracts were analyzed by WB with anti-NAP1 and anti-Tubulin antibodies.

C) CAL-1 NAP1$^{WT/WT}$ and CAL-1 NAP1 V84M$^{KI/KI}$ cells were incubated with a medium containing LyoVec alone (Mock) or transfected with 5'ppp-dsRNA using LyoVec (5'ppp-dsRNA). After 6 h, cells were fixed before staining with anti-pTBK1 or anti-NAP1 antibody. Representative confocal images (n=3). On microscopy images, square boxes have been magnified (3X) at the top left. Scale bars: 10 µm and 2 µm for insets.
D) Expression of 6 ISGs assessed by RT-qPCR in different clones of THP-1 with (WT/KI) (7 clones) or without (WT/WT) (3 clones) the mutation AZI2 p.V84M inserted by CRISPR-Cas9.
E) Expression of 2 ISGs assessed by flow cytometry in different clones of THP-1 with (WT/KI) or without (WT/WT) the mutation AZI2 p.V84M inserted by CRISPR-Cas9. **P < 0.01 (Wilcoxon test).
Source data are provided as a Source Data file.

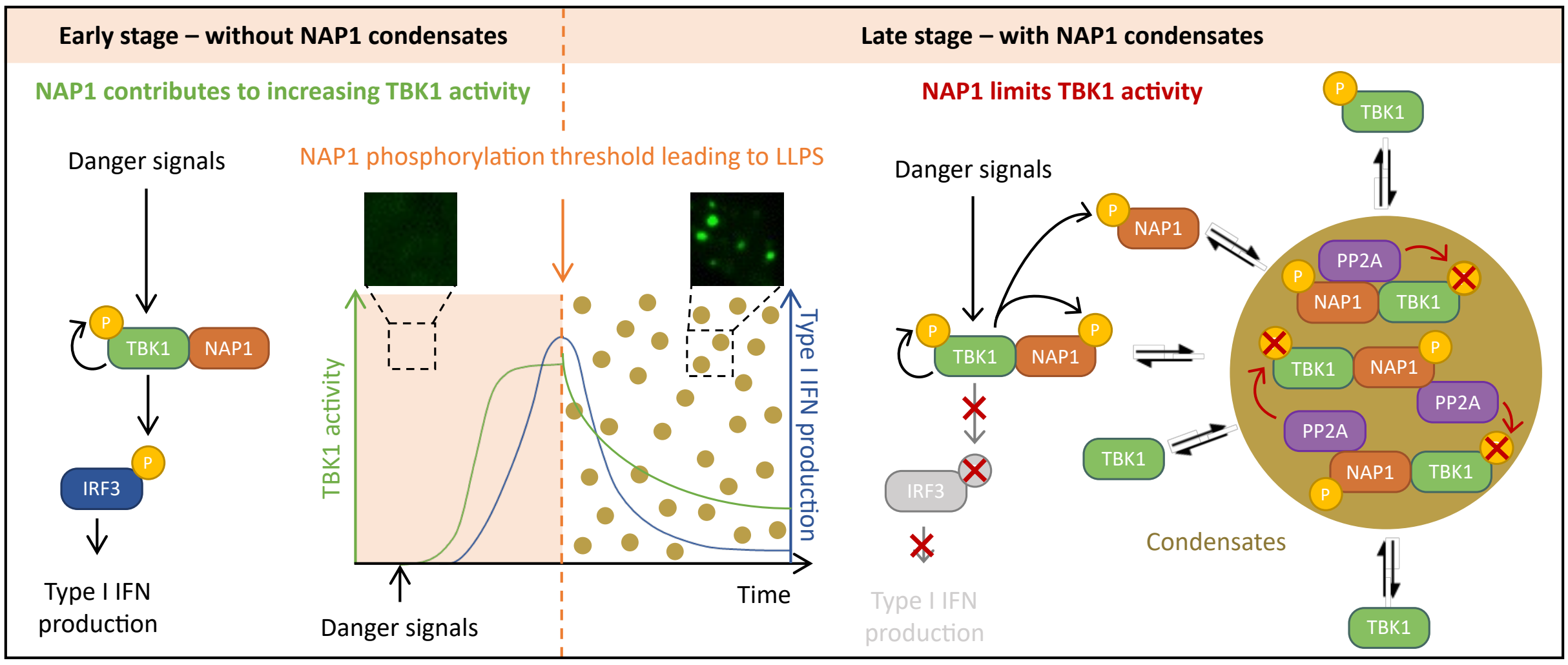

Supplementary Fig. 11 | A model for the dual role of NAP1 in the regulation of the IFN-β induction pathway

Upon cellular exposure to danger signals, NAP1 contributes to the activation of TBK1. Being a substrate of the kinase, NAP1 is also phosphorylated. When the amount of phosphorylated NAP1 reaches a critical threshold, it triggers the condensation of the adaptor protein, in which TBK1 and PP2A are concentrated, leading to the dephosphorylation of the kinase and its deactivation.

Supplementary Notes

Case reports

Patient A (AZI2: p.V84M)

Female patient followed for an undiagnosed suspected monogenic interferonopathy in France. The clinical presentation is polymorphic and multisystemic, with neurologic, gastrointestinal, pulmonary, hematologic, and immunologic involvement from early childhood.

Neurologically, the patient presents with longstanding spastic diplegia and a pyramidal syndrome, with brain MRI showing diffuse FLAIR and T2 hyperintensities in cortical white matter—predominantly frontal and temporal—as well as calcifications of the basal ganglia. In 2023, she developed a Devic-type neuromyelitis optica (NMO) associated with anti-AQP4 antibodies, presenting with headaches, photophobia. MRI confirmed panmyelitis, and CSF analysis showed hyperproteinorachia and hypoglycorachia without pleiocytosis. After the two first infusions of rituximab, associated with corticosteroid pulses the patient improved. B cells depletion was persistent at 1-year follow-up.

She has a long-standing profile of positive antinuclear antibodies, anti-DNA, and triple antiphospholipid positivity (anti-cardiolipin, anti-b2GP1 and lupus anticoagulant), as well as transient hemolytic anemia episodes, the latest of which occurred in 2022. Lymphocyte phenotyping showed no major quantitative deficits at followed by a persistent B-cell depletion post rituximab as well as a CD4+ and CD8+ T cell lymphopenia. High interferon levels have been consistently recorded since 2018 as well as high interferon signatures.

Pulmonary involvement includes an interstitial lung disease first documented in 2018, with progressive restriction on pulmonary function tests (CPT 69–76%) and a stable decrease in DLCO. Despite this, the patient remains clinically compensated with no oxygen requirement or desaturation on effort. Imaging has shown non-progressive lesions and no active infection, although past pneumococcal pneumonia and arthritis were documented. Current management includes close functional monitoring.

The first symptoms in early life were a transient lacerated gastritis, mild duodenitis and hepatitis. Liver biopsy showed cytolytic hepatitis with lymphocytic infiltration and minimal fibrosis. Hepatic elastography remains normal.

The therapeutic history includes multiple lines: azathioprine and hydroxychloroquine, prolonged corticosteroids, mycophenolate mofetil, aspirin with interruptions due to hematomas, and rituximab. Given the complex autoimmune and inflammatory profile, maintenance treatment with low-dose corticosteroids is currently pursued.

Despite multisystem involvement, the patient maintain stable pulmonary function and neurological status. The overall evolution is consistent with a likely monogenic interferonopathy.

Patient B (AZI2 : p.K109E)

This female patient presented with idiopathic thrombocytopenic purpura (ITP) at the age of 3 years, which required corticosteroid therapy due to the ineffectiveness of immunoglobulins. The etiological workup revealed positive antinuclear antibodies (ANA) with a homogeneous and speckled pattern at a titer of 1:2500, while anti-DNA and anti-ENA antibodies were negative.

Six months later, she was tested positive for IgG anticardiolipin antibodies (49 UI/l) and anti-beta2 glycoprotein I antibodies (48 UI/l), without the presence of lupus anticoagulant. Annual follow-ups continued to show positive ANA (titer 1:5000) and elevated levels of IgG anticardiolipin (56 UI/ml) and anti-beta2 glycoprotein I antibodies (61 UI/l), with normal complement levels and no renal involvement.

At the age of 5, following sun exposure, she developed a malar erythematous rash, potentially indicative of discoid lupus, which resolved favorably. She also reported migratory joint pain affecting the cervical spine, upper limbs, knees, and ankles, predominantly in the morning and sometimes

causing insomnia, but without any physical limitations or arthritis. Hydroxychloroquine was started together with aspirin.

In the absence of new symptoms or relapse, aspirin and hydroxychloroquine were discontinued at the ages of 12 and 14 years old, respectively.

Autoimmune markers normalized except for persistently positive ANA without specificity and the absence of symptoms suggestive of a connective tissue disease flare.

At the age of 17 years old she is now asymptomatic and off therapy.

Patient C (AZI2: p.P302H)

Female patient affected by Down syndrome who developed a complex autoimmune profile combining systemic lupus erythematosus (SLE), type I autoimmune hepatitis (AIH), and intestinal autoimmunity.

At age 13, the patient presented with pancytopenia. ANA were strongly positive (>1:1280, speckled pattern), with low-titer anti-dsDNA antibodies. Bone marrow analysis was normal, and macrophage activation syndrome was ruled out. High-dose corticosteroid therapy (1 mg/kg) and hydroxychloroquine were initiated based on the probable diagnosis of SLE, leading to partial hematologic improvement.

Corticosteroid therapy was complicated by induced diabetes managed by insulinotherapy.

At age 14, the patient was diagnosed with type I AIH at the cirrhotic stage. Liver biopsy revealed portal fibrosis, lymphoplasmacytic infiltrates, hepatocellular disorganization, and features consistent with AIH. Autoimmune serology showed positive anti-actin (smooth muscle) antibodies (1:100), ANA (1:800, speckled and homogeneous) and pANCA positivity against MPO. Imaging (MRI, 2016) was consistent with liver fibrosis with early signs of portal hypertension.

Endoscopic evaluations between ages 14 and 17 revealed progressive esophageal varices and portal hypertensive gastropathy, requiring several band ligation procedures. Follow-up imaging at age 16 showed liver dysmorphia with increased splenomegaly and signs of collateral circulation. The last abdominal ultrasound at age 17 confirmed chronic liver changes without suspicious nodules.

Probable autoimmune thyroiditis was suspected during follow-up, though thyroid function tests remained normal, including the last panel at age 17. Two colonoscopies (ages 16 and 17) showed lymphocytic colitis and increased polytypic plasma cells, consistent with autoimmune intestinal involvement.

The therapeutic course included corticosteroids (initiated at age 13 and tapered off by age 17), hydroxychloroquine (stopped at age 14), and azathioprine. This case illustrates a striking constellation of autoimmune disorders—lupus, autoimmune hepatitis and intestinal autoimmunity.

Supplementary reference

1. Piovesan, D. *et al.* MOBIDB in 2025: integrating ensemble properties and function annotations for intrinsically disordered proteins. *Nucleic Acids Res.* **53**, D495–D503 (2025).
