# Supplementary material for: NAP1 switches from an activator to a limiter of interferon induction by trapping TBK1 in condensates
Source: Nat Commun. 2026 Aug 19;17:9915. doi: 10.1038/s41467-026-76769-w (PMC13582825; doi:10.1038/s41467-026-76769-w)
Supplement: Supplementary file 2 [file 41467_2026_76769_MOESM2_ESM.pdf]

nature portfolio

Corresponding author(s): Yves Gaudin and Damien Glon

Last updated by author(s): Jul 15, 2026

Reporting Summary

Nature Portfolio wishes to improve the reproducibility of the work that we publish. This form provides structure for consistency and transparency in reporting. For further information on Nature Portfolio policies, see our Editorial Policies and the Editorial Policy Checklist.

Statistics

For all statistical analyses, confirm that the following items are present in the figure legend, table legend, main text, or Methods section.

n/a	Confirmed	
☐	☒	The exact sample size (*n*) for each experimental group/condition, given as a discrete number and unit of measurement
☐	☒	A statement on whether measurements were taken from distinct samples or whether the same sample was measured repeatedly
☐	☒	The statistical test(s) used AND whether they are one- or two-sided<br>*Only common tests should be described solely by name; describe more complex techniques in the Methods section.*
☐	☒	A description of all covariates tested
☒	☐	A description of any assumptions or corrections, such as tests of normality and adjustment for multiple comparisons
☐	☒	A full description of the statistical parameters including central tendency (e.g. means) or other basic estimates (e.g. regression coefficient) AND variation (e.g. standard deviation) or associated estimates of uncertainty (e.g. confidence intervals)
☐	☒	For null hypothesis testing, the test statistic (e.g. *F*, *t*, *r*) with confidence intervals, effect sizes, degrees of freedom and *P* value noted<br>*Give P values as exact values whenever suitable.*
☒	☐	For Bayesian analysis, information on the choice of priors and Markov chain Monte Carlo settings
☒	☐	For hierarchical and complex designs, identification of the appropriate level for tests and full reporting of outcomes
☒	☐	Estimates of effect sizes (e.g. Cohen's *d*, Pearson's *r*), indicating how they were calculated

Our web collection on statistics for biologists contains articles on many of the points above.

Software and code

Policy information about availability of computer code

Data collection	RT-qPCR data were collected using CFX96 Manager, confocal microscopy images were collected using Leica LAS X, live imaging were performed on Metamorph, WB were acquired on Odyssey, luciferase activities were measured on Flo12 Sirus Software
Data analysis	Softwares used : Prism (version 11), Leica LAS X, FIJI/ImageJ (version 2.16), AlphaFold3 and PyMOL (version 3.1.4.1)

For manuscripts utilizing custom algorithms or software that are central to the research but not yet described in published literature, software must be made available to editors and reviewers. We strongly encourage code deposition in a community repository (e.g. GitHub). See the Nature Portfolio guidelines for submitting code & software for further information.

Data

Policy information about availability of data

All manuscripts must include a data availability statement. This statement should provide the following information, where applicable:

- Accession codes, unique identifiers, or web links for publicly available datasets
- A description of any restrictions on data availability
- For clinical datasets or third party data, please ensure that the statement adheres to our policy

All data necessary to support the conclusions of this study are included in the article and the Supplementary Information. Source data are provided with this paper. The mass spectrometry dataset generated in this study have been deposited in the PRIDE database under accession code PXD062298.

Research involving human participants, their data, or biological material

Policy information about studies with human participants or human data. See also policy information about sex, gender (identity/presentation), and sexual orientation and race, ethnicity and racism.

Reporting on sex and gender	N/A
Reporting on race, ethnicity, or other socially relevant groupings	N/A
Population characteristics	N/A
Recruitment	N/A
Ethics oversight	Human samples are part of INSERM UMR1163/Imagine collection declared to the French Ministere de la Recherche (CODECOH no DC-2020-3994). Before the study, all patients signed informed consents approved by the CERAPH-Centre (IRB: #00011928).

Note that full information on the approval of the study protocol must also be provided in the manuscript.

Field-specific reporting

Please select the one below that is the best fit for your research. If you are not sure, read the appropriate sections before making your selection.

[x] Life sciences [ ] Behavioural & social sciences [ ] Ecological, evolutionary & environmental sciences

For a reference copy of the document with all sections, see nature.com/documents/nr-reporting-summary-flat.pdf

Life sciences study design

All studies must disclose on these points even when the disclosure is negative.

Sample size	For all the experiments we performed at least three independant biological replicates (the n values are inicated in each legend figures).
Data exclusions	No data were excluded from the analysis.
Replication	For all the experiments we performed at least three independant biological replicates.
Randomization	No randomization was required.
Blinding	N/A

Reporting for specific materials, systems and methods

We require information from authors about some types of materials, experimental systems and methods used in many studies. Here, indicate whether each material, system or method listed is relevant to your study. If you are not sure if a list item applies to your research, read the appropriate section before selecting a response.

Materials & experimental systems

n/a	Involved in the study	
[ ]	[x]	Antibodies
[ ]	[x]	Eukaryotic cell lines
[x]	[ ]	Palaeontology and archaeology
[x]	[ ]	Animals and other organisms
[ ]	[x]	Clinical data
[x]	[ ]	Dual use research of concern
[x]	[ ]	Plants

Methods

n/a	Involved in the study	
[x]	[ ]	ChIP-seq
[x]	[ ]	Flow cytometry
[x]	[ ]	MRI-based neuroimaging

Antibodies

Antibodies used	Primary antibodies used in this study included mouse monoclonal anti-Actin-β (Millipore, #MAB1501) used at a 1:10 000 dilution for WB analysis; mouse monoclonal anti-α-Tubulin (Sigma, #T6199) used at a 1:1000 dilution for WB analysis; mouse monoclonal anti-FLAG (Cell Signaling Technology, #8146) used at a 1:1000 dilution for WB analysis, at a dilution 1:100 for IF analysis and at a 1:100 dilution for IP analysis ; rabbit monoclonal anti-FLAG (Cell Signaling Technology, #14793) used at a 1:1000 dilution for WB analysis and at a 1:100 dilution for IF analysis; rabbit monoclonal anti-GAPDH (Cell Signaling Technology, #2118) used at a 1:1000 dilution for WB analysis; mouse monoclonal anti-GFP (Roche, #11814460001) used at a 1:1000 dilution for WB analysis and a 1:50 dilution for IP

analysis; monoclonal mouse anti-HA (Abcam, #ab130275) used at a 1:100 dilution for IP analysis ; rabbit polyclonal anti-IRF3 (Santa Cruz, #sc-9082) used at a 1:1000 for WB analysis and a 1:100 dilution for IF analysis ; rabbit polyclonal anti-MAVS (Bethyl, #A300-782A) used at a 1:1000 dilution for WB analysis ; rabbit monoclonal anti-mCherry (Cell Signaling Technology, #43590) used at a 1:1000 dilution for WB analysis ; rabbit monoclonal anti-MYC (Cell Signaling Technology, #2278) used at a 1:1000 dilution for WB analysis ; mouse monoclonal anti-RABV-N (#81C4, previously described60) used at a 1:2500 dilution for IF analysis ; rabbit monoclonal anti-NAP1 (Abcam, #192253) used at a 1:1000 dilution for WB analysis and a 1:100 dilution for IF analysis ; rabbit monoclonal anti-NAP1 (Proteintech, #15042-1-AP) used at a 1:1000 dilution for WB analysis and a 1:900 dilution for IF analysis ; mouse monoclonal anti-NAP1 (Proteintech, #68316-1) used at a 1:1000 dilution for IF analysis ; mouse monoclonal anti-RABV-P (#26G6, previously described61 used at a 1:1000 dilution for WB analysis ; rat monoclonal anti-PP2A-Aα (Santa Cruz ; #sc-56953) used at a 1:100 dilution for IF analysis ; rabbit monoclonal anti-PP2A-C (Cell Signaling Technology ; #2259S) used at a 1:1000 dilution for WB analysis ;mouse monoclonal anti-RIG-I (Santa Cruz, #sc-376845) used at a 1:1000 dilution for WB analysis ; rabbit monoclonal anti-SINTBAD (Cell Signaling Technology, #8605) used at a 1:1000 dilution for WB analysis ; rabbit polyclonal anti-SINTBAD (Abcam, #ab106394) used at a 1:200 dilution for IF analysis ; mouse monoclonal anti-TANK (Santa Cruz, #sc-166642) used at a 1:1000 dilution for WB analysis and a 1:100 dilution for IF analysis ; rabbit monoclonal anti-TBK1 (Abcam, #ab40676) used at a 1:1000 dilution for WB analysis and a 1:250 dilution for IF analysis ; rabbit monoclonal anti-phospho-TBK1 (Ser172) (Cell Signaling Technology, #5483) used at a 1:1000 dilution for WB analysis and a 1:250 dilution for IF analysis and mouse monoclonal anti-V5 (Invitrogen, #R960-25) used at a 1:5000 dilution for WB analysis.
Secondary antibodies used for IF analysis in this study were purchased from Invitrogen and included Alexa 488 goat anti-rabbit IgG (#A-11008); Alexa 488 goat anti-mouse IgG (#A-11029); Alexa 488 goat anti-rat IgG (#A11006); Alexa 568 goat anti-mouse IgG (#A-11031) and Alexa 647 goat anti-rabbit IgG (#A-21246).
Secondary antibodies used for WB analysis in this study included anti-rabbit 800-conjugated IgG (Cell Signaling Technology, #5151), anti-mouse 680-conjugated IgG (Cell Signaling Technology, #5470), goat anti-rabbit IgG-HRP (Invitrogen, #31460) and goat anti-mouse IgG-HRP (Sigma, #A4416).

Validation: Validation statements from the antibodies used can be found in the manufacturers websites.

Eukaryotic cell lines

Policy information about cell lines and Sex and Gender in Research

Cell line source(s)	Human (HEK293T, HAP1, THP-1 and CAL-1 cells - WT and KO cells).
Authentication	Commercially available (ATCC or Horizon) and others cell lines have been generated for this study.
Mycoplasma contamination	All cell lines were regularly tested for mycoplasma contamination and tested negative.
Commonly misidentified lines (See ICLAC register)	No commonly misidentifed cell lines were used in this study.

Clinical data

Policy information about clinical studies

All manuscripts should comply with the ICMJE guidelines for publication of clinical research and a completed CONSORT checklist must be included with all submissions.

Clinical trial registration	French Ministère de la Recherche (CODECOH no DC-2020-3994)
Study protocol	Informed consents were approved by the CERAPH-Centre (IRB: #00011928)
Data collection	INSERM UMR1163/Imagine Collection
Outcomes	Identification of variants associated with interferonopathies

Plants

Seed stocks	N/A
Novel plant genotypes	N/A
Authentication	N/A
