# Supplementary material for: NAP1 switches from an activator to a limiter of interferon induction by trapping TBK1 in condensates
Source: Nat Commun. 2026 Aug 19;17:9915. doi: 10.1038/s41467-026-76769-w (PMC13582825; doi:10.1038/s41467-026-76769-w)
Supplement: Supplementary file 3 [file 41467_2026_76769_MOESM3_ESM.pdf]

nature portfolio

Peer Review File

NAP1 switches from an activator to a limiter of interferon induction by trapping TBK1 in condensates

Corresponding Author: Dr Yves GAUDIN

This file contains all reviewer reports in order by version, followed by all author rebuttals in order by version.

Version 0:

Reviewer comments:

Reviewer #1

(Remarks to the Author)
The paper by Glon et al. describe a mechanism by which NAP1 controls type I interferon (IFN) signaling in a multi-step process which involves formation of condensates containing NAP1 and TBK1, a kinase essential the activation of IFN and NF-κB signaling pathways. Phosphorylation of NAP1 by TBK1 promotes the recruitment of PP2A into these condensates, leading to dephosphorylation of TBK1 and termination of IFN signaling. In addition, the authors identify three heterozygous NAP1 variants in patients with lupus and interferonopathies and demonstrate in heterologous cell models that these variants fail to form condensates, causing uncontrolled IFN signaling. The study provides novel insight into mechanisms that terminate type I IFN signaling and therefore addresses an important scientific question. However, the current data are not sufficient to support the proposed conclusions and require additional experimental validation. In particular, the causal role of NAP1 variants in human type I IFN-driven diseases remains unclear.

Specific comments
Line 116. The authors show that while TBK1 can form condensates in NAP1 knockout cells, their number is decreased compared to wild type cells. On the other hand, TBK1 knockout cells contained fewer endogenous NAP1 condensates. Given the important role of NAP1 in mitosis (Paul et. al. JCB 2023) and in nucleosome assembly (Andrews et al. Mol Cell 2010), the observed effects on condensate formation may reflect defects in cell integrity and viability. What were the cellular phenotypes of the NAP1 (and other adaptor) knockout cells regarding proliferation and activation of immune signaling pathways such as NF-kB or IFN both at steady state and in response to stimulation?

Line 131. No adaptor protein condensates were observed in MAVS-deficient cells following 5′ppp-dsRNA stimulation, whereas diABZI treatment induced the formation of TBK1 condensates. Did diABZI also trigger the formation of NAP1–TBK1 condensates, and was this process similarly impaired in STING-deficient cells?

Line 159. The authors convincingly demonstrate the liquid-like properties of TBK1 adaptor proteins using overexpressed GFP-tagged constructs. However, it remains essential to demonstrate co-condensation of TBK1 with NAP1 and/or other adaptor proteins. Importantly, these findings should also be validated at the level of endogenous proteins.

Line 208. The functional roles of SINTBAD and TANK condensates, which appear to be distinct from the role of NAP1 condensates, remains unclear. How do they influence NAP1-TBK1 condensates and IFN signaling?

Line 237 and line 289. The dual effect of NAP1 on TBK1-dependent IFN activation is intriguing. However, all experiments addressing this point were performed under overexpression conditions. Validation in a more physiological setting is needed, for example by using cell models with knock-in NAP1 mutants that are unable to interact with TBK1 or undergo TBK1-dependent phosphorylation.

Line 336. NAP1 phosphorylation by TBK1 was only demonstrated in cells overexpressing tagged NAP1 and TBK. Again, this should be confirmed on a physiological level using cell models with knock-in NAP1 mutants that are unable to interact with TBK1 or undergo TBK1-dependent phosphorylation.

Line 395. The conclusion that TBK1 is dephosphorylated by PP2A is based on indirect experimental evidence and requires direct validation. If PP2A is indeed essential for termination of TBK1-dependent IFN signaling, then PP2A knockdown or

knockout should result in uncontrolled IFN activation.

Line 417. The description of the human NAP1 variants in Fig. S7 requires clarification. Although rare, both K109E and V84M are reported in the gnomAD database. Based on the discussion, it appears that all heterozygous variants were inherited from healthy parents. Were extended family members examined for clinical signs of disease and for segregation with the NAP1 variants? While the case reports outline the clinical manifestations, it remains unclear whether these patients exhibit a detectable interferon signature. Moreover, what is the degree of evolutionary conservation of the affected amino acid residues across species? Functional analyses of the NAP1 variants were performed in overexpression systems. To substantiate that these variants impair NAP1–TBK1 condensation and drive uncontrolled IFN signaling, validation in patient-derived cells or in gene-edited models is needed. At present, there is neither sufficient genetic nor functional evidence to establish a causal link between NAP1 variants and disease.

Line 457. The term “dominant effect” requires clarification. Does this refer to the mode of inheritance or to the functional impact of the variants? Neither dominant inheritance nor segregation of the NAP1 variants within families has been demonstrated. Moreover, all functional studies were performed using mutant NAP1 overexpression in cells with endogenous wildtype NAP1. If the identified variants impair condensate formation, they would abrogate NAP1 function and thus behave as loss-of-function mutations, rather than dominant-negative alleles.

Reviewer #2

(Remarks to the Author)
The study titled "NAP1 switches from an activator to a limiter of interferon induction by trapping TBK1 in condensates" reveals a novel regulatory mechanism in the type I interferon induction pathway, characterized the condensates formed by the three TBK1-adaptor proteins. Thus, this study could provid a pathophysiological mechanism in patients with interferonopathies or lupus. In order to fully illustrate the function of the condensates formed by TBK1 and others, the following questions need to be addressed.

Major points:
1.While multiple phosphorylation sites on NAP1 were identified, the authors mentioned that no single site is solely responsible for condensate formation. How exactly do these phosphorylation sites collectively influence LLPS? Is it purely charge-based, or are there structural determinants?
2.Many experiments rely on overexpression systems. To what extent do the observed condensates and regulatory dynamics reflect endogenous protein levels and physiological conditions?
3.The study is primarily based on cell line models. How do these mechanisms operate in vivo, especially in immune cells relevant to interferonopathies and lupus? While patient mutations were identified, functional validation was done in engineered cell lines. Would primary cells from patients show the same defects in condensate formation and IFN regulation?
4.Could targeting NAP1 condensates or their components (e.g., PP2A) offer a therapeutic strategy for interferonopathies or lupus?

Minor points:
1.Is there any particularity in the localization of these condensates within the cells? For example, whether it is close to a certain organelle?
2.What does the the fusion events mentioned at Fig.2C represent? Under what circumstances would the fusion of condensates occur?
3.The author mentioned in Fig.2 that NAP1 and TANK formed distinct condensates. However, no peaks without co-localization were observed in Fig.2F with co-transfection mC-SINTBAD and GFP-TANK, considering some of SINTBAD should form condensates with NAP1 without TANK.
4.Do these three mutations obtained from patients have any effect on the phosphorylation of NAP1? Does it have any impact on the interaction between SINTBAD and NAP1?

Version 1:

Reviewer comments:

Reviewer #1

(Remarks to the Author)
The revised manuscript addresses many of the mechanistic concerns raised during the initial review. The additional experiments provide further support for a role of NAP1 condensates in regulating TBK1-dependent interferon signaling. Nevertheless, some aspects of the proposed mechanism remain based on indirect evidence, particularly regarding endogenous NAP1 phosphorylation and the precise role of PP2A in TBK1 deactivation. These limitations should be discussed more explicitly.

My major remaining concern relates to the human genetics section.
The authors provide additional functional evidence showing that the identified AZI2 variants impair NAP1 condensate formation and alter interferon signaling in cellular systems. However, these data do not establish that AZI2 is a disease-causing gene. The variants are inherited from unaffected parents where information is available, segregation data are

lacking, extended family studies could not be performed, and only a very limited number of affected individuals have been identified. Thus, the genetic evidence remains insufficient to support causality.
Importantly, if AZI2 cannot currently be considered a bona fide disease gene, then the clinical phenotypes observed in the reported individuals cannot be interpreted as evidence that AZI2 deficiency causes interferonopathy, lupus, or the associated clinical manifestations. While the patient descriptions are informative, they remain clinical observations associated with candidate variants of uncertain pathogenic significance. Although the functional studies demonstrate effects on NAP1 biology, they do not establish causality and therefore cannot demonstrate that these variants are responsible for the patients' clinical phenotypes.

Accordingly, I recommend substantial downtoning of the human genetics claims throughout the manuscript. In particular, statements implying that the identified variants provide a molecular explanation for interferonopathies, define a novel monogenic disorder, or explain the observed patient phenotypes are not sufficiently supported by the available evidence. As causality has not been established, the reported clinical manifestations cannot currently be interpreted as consequences of AZI2 variation. The data support disruption of NAP1 biology by these variants, but do not demonstrate that they are disease-causing in the reported patients.

Reviewer #2

(Remarks to the Author)
The concerns I raised have been well addressed.

Version 2:

Reviewer comments:

Reviewer #1

(Remarks to the Author)
The authors have fully addressed my concerns.

Open Access This Peer Review File is licensed under a Creative Commons Attribution 4.0 International License, which permits use, sharing, adaptation, distribution and reproduction in any medium or format, as long as you give appropriate credit to the original author(s) and the source, provide a link to the Creative Commons license, and indicate if changes were made.
In cases where reviewers are anonymous, credit should be given to 'Anonymous Referee' and the source.
The images or other third party material in this Peer Review File are included in the article’s Creative Commons license, unless indicated otherwise in a credit line to the material. If material is not included in the article’s Creative Commons license and your intended use is not permitted by statutory regulation or exceeds the permitted use, you will need to obtain permission directly from the copyright holder.
To view a copy of this license, visit https://creativecommons.org/licenses/by/4.0/

REBUTTAL

Reviewer #1

The paper by Glon et al. describe a mechanism by which NAP1 controls type I interferon (IFN) signaling in a multi-step process which involves formation of condensates containing NAP1 and TBK1, a kinase essential the activation of IFN and NF-κB signaling pathways. Phosphorylation of NAP1 by TBK1 promotes the recruitment of PP2A into these condensates, leading to dephosphorylation of TBK1 and termination of IFN signaling. In addition, the authors identify three heterozygous NAP1 variants in patients with lupus and interferonopathies and demonstrate in heterologous cell models that these variants fail to form condensates, causing uncontrolled IFN signaling. The study provides novel insight into mechanisms that terminate type I IFN signaling and therefore addresses an important scientific question. However, the current data are not sufficient to support the proposed conclusions and require additional experimental validation. In particular, the causal role of NAP1 variants in human type I IFN-driven diseases remains unclear.

We sincerely thank the reviewer for their insightful and constructive comments.

We are confident that the additional experiments we have conducted, which are included in the revised manuscript, will address most of their concerns and provide robust support for our conclusions.

Specific comments:

Line 116. The authors show that while TBK1 can form condensates in NAP1 knockout cells, their number is decreased compared to wild type cells. On the other hand, TBK1 knockout cells contained fewer endogenous NAP1 condensates. Given the important role of NAP1 in mitosis (Paul et. al. JCB 2023) and in nucleosome assembly (Andrews et al. Mol Cell 2010), the observed effects on condensate formation may reflect defects in cell integrity and viability. What were the cellular phenotypes of the NAP1 (and other adaptor) knockout cells regarding proliferation and activation of immune signaling pathways such as NF-kB or IFN both at steady state and in response to stimulation?

We thank the reviewer for this thoughtful comment.

It is indeed well established that NAP1 and TBK1 both play roles in mitosis, but we would like to clarify that the Nap1 protein (Nucleosome Assembly Protein 1, encoded by the *NAP1* gene) involved in nucleosome assembly is distinct from NAP1 (NAK-associated Protein 1, encoded by the *AZI2* gene), which is the focus of our study.

To address this point, we evaluated the impact of TBK1 and adaptor knockout on cell proliferation in HEK293T and HAP1 cell lines (**new Fig. S2C,** lines 123-127). We observed only a slight growth delay in $TBK1^{KO}$ cells, while NAP1 and other adaptors had no significant impact.

Moreover, basal levels of IFNβ transcripts were comparable between TBK1-$adaptor^{KO}$ and parental cell lines (**new Fig. S5E,** lines 266-267).

Finally, knockout of TBK1-adaptor proteins did not alter NF-κB signaling, either at steady state (**new Fig. S5F**, lines 267-269) or upon TNFα stimulation (**new Fig. S5G,** lines 267-271). Nevertheless, in $NAP1^{KO}$ HAP1 cells, we observed a slight but significant increase in NF-κB signaling upon TNFα-induction compared to parental HAP1 cells.

Line 131. No adaptor protein condensates were observed in MAVS-deficient cells following 5'ppp-dsRNA stimulation, whereas diABZI treatment induced the formation of TBK1 condensates. Did diABZI

also trigger the formation of NAP1–TBK1 condensates, and was this process similarly impaired in STING-deficient cells?

We have now assessed the formation of condensates for all TBK1 adaptors in response to diABZI stimulation in THP-1 cells (naturally expressing STING) and STING-deficient HEK293T cells, either transfected or not with a plasmid encoding STING.

Under these conditions, we observed condensate formation for TBK1, NAP1, and SINTBAD, but not for TANK (**new Figs. S2E and S2F**, new paragraph lines 143-152).

Line 159. The authors convincingly demonstrate the liquid-like properties of TBK1 adaptor proteins using overexpressed GFP-tagged constructs. However, it remains essential to demonstrate co-condensation of TBK1 with NAP1 and/or other adaptor proteins. Importantly, these findings should also be validated at the level of endogenous proteins.

We fully agree with the reviewer that this was a notable omission in our manuscript. We now present confocal microscopy images of cells infected with rabies virus, demonstrating that endogenous TBK1 and NAP1 colocalize within the same condensates, whereas SINTBAD and TANK form distinct condensates separate from those containing TBK1 (**new Fig. 2D**, new paragraph lines 188-191).

Line 208. The functional roles of SINTBAD and TANK condensates, which appear to be distinct from the role of NAP1 condensates, remains unclear. How do they influence NAP1-TBK1 condensates and IFN signaling?

We appreciate the reviewer's interest in the functional roles of SINTBAD and TANK condensates. In the previous version of the manuscript, we acknowledged this as a limitation in the Discussion, stating: "*This study of course has limitations. We only identified the role of NAP1 condensates but did not identify any function associated with those formed by SINTBAD and TANK. Since these adaptor proteins also undergo LLPS and interact with TBK1, their potential contribution to the regulation of the IFN pathway or any other pathway in which TBK1 is involved remains an open question.*" We keep this paragraph in the revised version (lines 573-577).

However, as the functional exploration of SINTBAD and TANK condensates falls beyond the scope of our current study, which focuses on NAP1, and given that we observed no impact of SINTBAD or TANK knockout on IFN induction, we did not investigate their influence on NAP1-TBK1 condensates or IFN signaling further. We believe this question would be better addressed in a dedicated study, where it can be explored in depth without diverting from the central focus of this work.

Line 237 and line 289. The dual effect of NAP1 on TBK1-dependent IFN activation is intriguing. However, all experiments addressing this point were performed under overexpression conditions. Validation in a more physiological setting is needed, for example by using cell models with knock-in NAP1 mutants that are unable to interact with TBK1 or undergo TBK1-dependent phosphorylation.

We agree with this reviewer's remark. To address this point, we directly assessed the dual role of NAP1 using VSV-infected cells, avoiding any protein overexpression. By quantifying IFNβ transcript levels at multiple time points post-infection, we found that NAP1 knockout HEK293T cells displayed reduced IFNβ transcript levels at early time points (2 hours post-infection, h.p.i.), whereas these levels became higher than in the parental cell line at later stages (4 and 6 h.p.i.) (**new Fig. 4K,** new paragraph lines 308-314).

Line 336. NAP1 phosphorylation by TBK1 was only demonstrated in cells overexpressing tagged NAP1

and TBK. Again, this should be confirmed on a physiological level using cell models with knock-in NAP1 mutants that are unable to interact with TBK1 or undergo TBK1-dependent phosphorylation.

We agree with the reviewer's comment and appreciate their suggestion.

To address this point, we analyzed endogenous NAP1 phosphorylation in TBK1^{KO} HEK293T cells and the parental line by Western blot. However, we encountered a technical limitation: the commercial antibody we used detects a contaminating protein that migrates at the expected position of phosphorylated NAP1 (Fig. S2B). This issue does not arise when we express mCherry-tagged NAP1 as it migrates with higher molecular weight (72 kDa vs 45 kDa) or when we overexpress untagged NAP1, as the NAP1 bands (phosphorylated or not) are significantly more intense than the contaminant band (e.g., Fig. 4G).

Furthermore, generating the knock-in (KI) NAP1 mutants suggested by the reviewer would not resolve this issue. As we previously demonstrated, NAP1 phosphorylation does not depend on its interaction with TBK1 (Fig. 6A). Additionally, the multiplicity of phosphorylation sites (some of which we have likely not yet identified) makes it impractical to generate a KI cell line for a non-phosphorylatable mutant.

This remains the only point we were unable to address experimentally. We hope the reviewer will understand these limitations.

Line 395. The conclusion that TBK1 is dephosphorylated by PP2A is based on indirect experimental evidence and requires direct validation. If PP2A is indeed essential for termination of TBK1-dependent IFN signaling, then PP2A knockdown or knockout should result in uncontrolled IFN activation.

We sought direct validation by generating a PP2A-deficient cell line through knockout of the PP2A catalytic subunit (PP2A-Cα). However, consistent with previously published findings (Götz et al. PNAS, 1998, PMID: 9770493 ; Ding et al. Stem Cell Research, 2023, PMID: 36724553), we were unable to achieve complete extinction of PP2A-Cα expression, suggesting that homozygous knockout is lethal. Nonetheless, one of our clones exhibited a ~50% reduction in PP2A-Cα expression (**new Fig. S7I**). Upon infection of this clone with RABV, we observed a significant enhancement in IFN induction compared to the wild-type cell line (**New Fig. S7J**).These results, included in the manuscript (new paragraph, lines 440-445), confirm that PP2A plays a critical role in terminating TBK1-dependent IFN signaling.

Line 417. The description of the human NAP1 variants in Fig. S7 requires clarification. Although rare, both K109E and V84M are reported in the gnomAD database. Based on the discussion, it appears that all heterozygous variants were inherited from healthy parents. Were extended family members examined for clinical signs of disease and for segregation with the NAP1 variants? While the case reports outline the clinical manifestations, it remains unclear whether these patients exhibit a detectable interferon signature. Moreover, what is the degree of evolutionary conservation of the affected amino acid residues across species? Functional analyses of the NAP1 variants were performed in overexpression systems. To substantiate that these variants impair NAP1–TBK1 condensation and drive uncontrolled IFN signaling, validation in patient-derived cells or in gene-edited models is needed. At present, there is neither sufficient genetic nor functional evidence to establish a causal link between NAP1 variants and disease.

We thank the reviewer for these thoughtful remarks.

Both variants are present in gnomAD v4.1: AZI2 p.V84M is reported 19 times (allele frequency around 10-5) and p.K109E once (allele frequency around 10-7). We have added a Figure displaying the Minor

Allele Frequency (MAF) and CADD score of for all gnomAD v4.1 variants, with the patients' variants highlighted (**New Fig. S8C**).

The heterozygous variants were inherited from healthy parents in two of the three cases; no information is available for the third. Updated pedigrees are provided in **new Fig. S8A**.

The apparent discrepancy between the in vitro deleterious effect of variants in genes involved in primary immune deficiencies and dysregulations (PIDDs) and the incomplete penetrance observed in heterozygous carriers is a well-recognized phenomenon in the field. It strongly suggests that disease onset in these so-called monogenic disorders is modulated by additional factors, including mono-allelic expression, environmental triggers, epigenetic modifications, somatic mutations, or digenic interactions, as we and others have previously reported (**see references 1 to 5 at the end of our answers to this reviewer**). Resolving the full genetic architecture of incomplete penetrance in PIDDs remains an active area of investigation. We wish to emphasize that the primary aim of this report is to define the dual role of AZI2/NAP1 in TBK1 regulation and not to provide an exhaustive characterization of a new monogenic disorder, which would clearly require additional biological material and dedicated studies.

The parents were indeed reported as clinically asymptomatic by the physicians following these patients. However, no parental biological material was available to us, precluding direct testing for a subclinical type I IFN signature or early autoimmune features. Extended family members were not examined for segregation, as no additional samples could be obtained.

Evolutionary conservation analysis was also added as a new Supplementary Figure (**New Fig. S8D**, commented on line 456). Residues 84 and 302 are conserved across species.

We have now extended our functional analyses. For Patient A (p.V84M), for whom biological material was available, we demonstrate a persistently elevated plasma interferon-alpha level, as well as an elevated ISG signature in all blood longitudinal samples assessed by NanoString technology (**New Fig. S8E**, each data point represents an independent sample). We additionally profiled 96 pro-inflammatory cytokines and chemokines in five longitudinal serum samples using the OLINK platform, revealing a marked and consistent elevation of multiple inflammatory mediators, including IFN-γ and CXCL10 (Heatmap provided to reviewers; this figure can be incorporated into the manuscript if deemed appropriate).

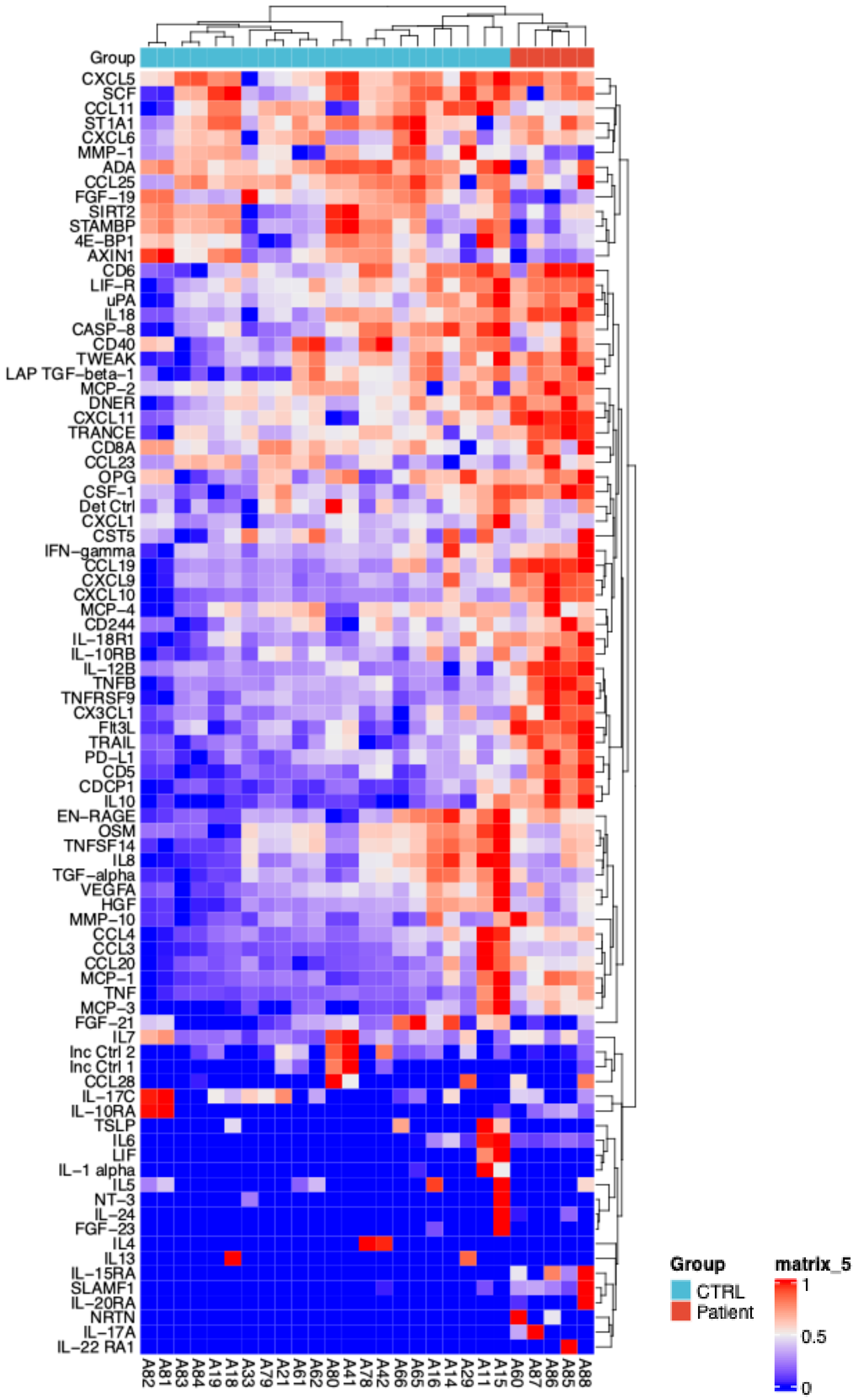

Finally, we have shown that the previously generated gene-edited cell lines, CAL-1 (pDC-like cell line) and THP-1 (monocyte-like cell line), harboring the p.V84M variant, exhibit elevated ISGs expression at steady state, at the mRNA level and at the protein level as assessed by flow-cytometry (**New Fig S9D and S9E**, lines 543-544). These experiments are providing orthogonal validation of the functional impact of this variant on NAP1–TBK1 condensation and IFN signaling.

Taken together, these results are consistent with a contributing role of the *AZI2* p.V84M variant in the clinical presentation of patient A, who exhibits a *bona fide* interferonopathy phenotype. We fully agree with the reviewer that formal proof of causality requires additional evidence, and we have accordingly revised the manuscript to avoid any claim that *AZI2* variants represent an established cause of a novel monogenic disorder.

Line 457. The term "dominant effect" requires clarification. Does this refer to the mode of inheritance or to the functional impact of the variants? Neither dominant inheritance nor segregation of the NAP1 variants within families has been demonstrated. Moreover, all functional studies were performed using mutant NAP1 overexpression in cells with endogenous wildtype NAP1. If the identified variants

impair condensate formation, they would abrogate NAP1 function and thus behave as loss-of-function mutations, rather than dominant-negative alleles.

We thank the reviewer for raising this important point.

In our manuscript, we used the expression "*dominant-negative effect*" to describe the molecular mechanism by which the NAP1 variants (V84M, K109E, P302H) disrupt the function of NAP1 WT. Our data showed that these mutants do not merely act as loss-of-function alleles but also impair the ability of WT NAP1 to form condensates (**Fig. 7I**) and to regulate TBK1 activity when WT and mutant are co-expressed in $NAP1^{KO}$ cells (**Fig. 7H**). Specifically:

The mutants still interact with TBK1 (**Fig. 7F**) and SINTBAD (**new Fig 7G**) but fail to form condensates efficiently (**Fig 7C**), leading to dysregulated IFN-β signaling.

In heterozygous conditions, the presence of a single mutant allele counteracts the inhibitory function of WT NAP1 (**Fig. 7H**), consistent with heterodimers of WT and mutant protomers behaving like mutant homodimers. This is the hallmark of a dominant-negative mechanism at the molecular level.

We acknowledge that dominant inheritance (in the genetic sense) has not been demonstrated, as we did not assess familial segregation. Our previous manuscript did not mention inheritance patterns and our functional data exclusively support a dominant-negative functional effect in the context of NAP1 condensate formation and IFN signaling regulation.

To avoid any ambiguity, we have revised the paragraph as follows:

"A key mechanistic insight is the apparent disruption of the ability of NAP1 mutants to form condensates, a feature essential for modulating TBK1 activity in both ectopic expression and knock-in models. Mutant proteins exhibited reduced condensate formation and higher critical concentration while still interacting with TBK1 and SINTBAD. Moreover, heterozygous expression of these variants was sufficient to counteract the inhibitory function of NAP1 WT, suggesting ***a dominant-negative mechanism at the molecular level****. In this scenario, heterodimers between WT and NAP1 mutants behave like mutant homodimers, likely rendering WT NAP1 unable to properly regulate TBK1 activity. These findings underscore the critical role of NAP1 condensates in immune regulation and suggest that AZI2 mutations could act as a genetic predisposition to immune dysregulation through* ***a dominant-negative functional impact****."* (Lines 543-553)

References

1. Gruber, C. & Bogunovic, D. Incomplete penetrance in primary immunodeficiency: a skeleton in the closet. *Hum Genet* **139**, 745–757 (2020).

2. Stewart, O. *et al.* Monoallelic expression can govern penetrance of inborn errors of immunity. *Nature* 1–12 (2025) doi:10.1038/s41586-024-08346-4.

3. Rieux-Laucat, F. & Casanova, J.-L. Immunology. Autoimmunity by haploinsufficiency. *Science* **345**, 1560–1561 (2014).

4. Hadjadj, J. *et al.* Early-onset autoimmunity associated with SOCS1 haploinsufficiency. *Nat Commun* **11**, 5341 (2020).

5. Jeanpierre, M. *et al.* Haploinsufficiency in PTPN2 leads to early-onset systemic autoimmunity from Evans syndrome to lupus. *J Exp Med* **221**, e20232337 (2024).

Reviewer #2

The study titled "NAP1 switches from an activator to a limiter of interferon induction by trapping TBK1 in condensates" reveals a novel regulatory mechanism in the type I interferon induction pathway, characterized the condensates formed by the three TBK1-adaptor proteins. Thus, this study could provide a pathophysiological mechanism in patients with interferonopathies or lupus. In order to fully illustrate the function of the condensates formed by TBK1 and others, the following questions need to be addressed.

We appreciate the reviewer's insightful and constructive comments. The additional experiments now incorporated into the revised manuscript are designed to address their concerns and provide support for our findings.

Major points:

1.While multiple phosphorylation sites on NAP1 were identified, the authors mentioned that no single site is solely responsible for condensate formation. How exactly do these phosphorylation sites collectively influence LLPS? Is it purely charge-based, or are there structural determinants?

As already discussed in the earlier version of our manuscript, LLPS is most likely driven by a charge-based mechanism involving specific protein domains in line with observations for other proteins capable of inducing phase separations (previous refs. 53,54 and new ref. 55, Valverde et al. Nat. Com. 2023, PMID: 37813838).

To further investigate, we used AlphaFold3 to model the structural impact of phosphorylation on NAP1, both alone and in complex with TBK1. Our predictions indicate that phosphorylation does not significantly alter the overall structure of NAP1 in these contexts.

2. Many experiments rely on overexpression systems. To what extent do the observed condensates and regulatory dynamics reflect endogenous protein levels and physiological conditions?

We fully agree with the reviewer's point.

However, we would like to highlight that a significant number of our experiments were already performed under physiologically relevant conditions, without protein overexpression (Figs. 1A, 1B, 1C, 4A, 7I, S2A, previous S2C now S2D, S5A, previous S8D now S9C).

We have now expanded this analysis with imaging data obtained on endogenous proteins, demonstrating that TBK1 condensates colocalize with those of NAP1 but remain distinct from those formed by SINTBAD and TANK (**new Fig. 2D**, new paragraph lines 188-191). We also show that diABZI induces condensate formation by endogenous NAP1 and SINTBAD adaptors (**new Fig. S2E**, lines 143-147). Moreover, we have characterized the dual effect of endogenous NAP1 on IFNβ gene transcription during VSV infection (**new Fig. 4K**, new paragraph lines 308-314), and further clarified PP2A's role under physiological conditions (**new Fig. S7J**, new paragraph lines 440-445).

Taken together, these results indicate that our observations do not arise from overexpression artefacts, but rather reflect a physiological process.

3.The study is primarily based on cell line models. How do these mechanisms operate in vivo, especially in immune cells relevant to interferonopathies and lupus? While patient mutations were identified,

functional validation was done in engineered cell lines. Would primary cells from patients show the same defects in condensate formation and IFN regulation?

We thank the reviewer for raising this important point.

The primary objective of this manuscript is to establish a novel role for NAP1 in the regulation of TBK1 activity. The identification of rare variants of unknown significance in patients with dysregulated IFN production (interferonopathy and systemic lupus) provides additional, clinically grounded, support for this dual regulatory function of NAP1, rather than constituting the central claim of the study. A comprehensive characterization of these variants as a cause of a new monogenic disorder would require a dedicated investigation and, ideally, access to additional biological material.

That said, we were able to study primary cells from Patient A (p.V84M), for whom biological material was available, and the results are informative on several levels.

First, Patient A exhibits persistently elevated plasma IFN-α levels across multiple time points, as measured by single-molecule array (Simoa), along with a markedly increased interferon-stimulated gene score in resting PBMCs, assessed by NanoString technology (**New Fig. S8E**).

Second, longitudinal serum profiling by Olink technology across five independent samples revealed a broad and sustained elevation of pro-inflammatory cytokines and chemokines, including IFN-γ and the ISG-driven chemokine CXCL-10 (Figure provided to reviewer 1 above, this figure can be incorporated into the manuscript if deemed appropriate).

Third, and critically, knock-in THP-1 cells engineered to carry the p.V84M variant show spontaneous elevation of ISGs at the mRNA and protein level (**New Fig. S9D and S9E**, lines 499-500), providing orthogonal, cell-line-independent validation in a monocytic background that is directly relevant to innate immune responses.

Taken together, these data from both patient-derived primary cells and gene-edited myeloid cell lines support a contributing role of AZI2 p.V84M in the interferonopathy phenotype of Patient A, while acknowledging that the full extent of its pathogenic contribution warrants further exploration

4.Could targeting NAP1 condensates or their components (e.g., PP2A) offer a therapeutic strategy for interferonopathies or lupus?

Our manuscript characterizes the role of NAP1, in association with PP2A, in regulating interferon responses. Defects in this mechanism can indeed contribute to immune dysregulation. However, it remains unclear whether directly targeting this mechanism could serve as a viable therapeutic strategy. Restoring NAP1's ability to form condensates would likely be necessary for such an approach. At present, the most straightforward therapeutic strategy may still be to block the downstream effects of excess interferon production.

Minor points:

1.Is there any particularity in the localization of these condensates within the cells? For example, whether it is close to a certain organelle?

In our previous work (Scrima et al., 2023, Cell Rep.), we already showed that TBK1 condensates do not correspond to or localize with known organelles, including membrane organelles such as Golgi apparatus, ER, or mitochondria, as well as biomolecular condensates like stress granules.

In the present study, no specific localization was detected for the condensates of TBK1 and/or its adaptor proteins.

2.What does the the fusion events mentioned at Fig.2C represent? Under what circumstances would the fusion of condensates occur?

Fusion of liquid organelles formed via LLPS is generally a spontaneous, seemingly unregulated process observed when two liquid organelles of the same nature come into contact. However, specific cellular contexts or interactions with scaffolding proteins can modulate or even inhibit this process in certain cases. Investigating this aspect presents significant challenges, and we currently lack evidence suggesting that the fusion of TBK1-adaptor condensates is not spontaneous in the systems studied here.

3.The author mentioned in Fig.2 that NAP1 and TANK formed distinct condensates. However, no peaks without co-localization were observed in Fig.2F with co-transfection mC-SINTBAD and GFP-TANK, considering some of SINTBAD should form condensates with NAP1 without TANK.

We hypothesize that in the context of experiments where SINTBAD and TANK are overexpressed, endogenous NAP1 levels are insufficient to form condensates with SINTBAD from which TANK would be excluded.

4.Do these three mutations obtained from patients have any effect on the phosphorylation of NAP1? Does it have any impact on the interaction between SINTBAD and NAP1?

We previously showed that TBK1 also phosphorylates NAP1 variants, as assessed by the electrophoretic mobility shift of mutant proteins in gels (Fig. 7F). We now show that SINTBAD consistently interacts with NAP1 mutants (**new Fig. 7G**, line 479).

REBUTTAL

Reviewer #1

The revised manuscript addresses many of the mechanistic concerns raised during the initial review. The additional experiments provide further support for a role of NAP1 condensates in regulating TBK1-dependent interferon signaling. Nevertheless, some aspects of the proposed mechanism remain based on indirect evidence, particularly regarding endogenous NAP1 phosphorylation and the precise role of PP2A in TBK1 deactivation. These limitations should be discussed more explicitly.

We have included a paragraph in the last part of the discussion dedicated to the limitations of our study. We acknowledge that direct measurement of endogenous NAP1 phosphorylation under native conditions has not been performed (for reasons explained in our previous rebuttal letter). We also acknowledge that, while the colocalization, association, and inhibitor data for PP2A strongly support its role in TBK1 deactivation, the precise molecular mechanism remains to be defined.

Line 586-591 of the revised manuscript: *Furthermore, direct evidence for endogenous NAP1 phosphorylation under physiological conditions remains to be obtained. Although PP2A colocalizes with NAP1 in condensates, associates with NAP1, and its inhibition increases IFN-β signaling, the precise sequence of molecular events leading to TBK1 deactivation remains to be elucidated. Future studies will be needed to define the endogenous phosphorylation dynamics of NAP1 and the biochemical mechanism by which PP2A mediates TBK1 inactivation.*

My major remaining concern relates to the human genetics section. The authors provide additional functional evidence showing that the identified AZI2 variants impair NAP1 condensate formation and alter interferon signaling in cellular systems. However, these data do not establish that AZI2 is a disease-causing gene. The variants are inherited from unaffected parents where information is available, segregation data are lacking, extended family studies could not be performed, and only a very limited number of affected individuals have been identified. Thus, the genetic evidence remains insufficient to support causality.

Importantly, if AZI2 cannot currently be considered a bona fide disease gene, then the clinical phenotypes observed in the reported individuals cannot be interpreted as evidence that AZI2 deficiency causes interferonopathy, lupus, or the associated clinical manifestations. While the patient descriptions are informative, they remain clinical observations associated with candidate variants of uncertain pathogenic significance. Although the functional studies demonstrate effects on NAP1 biology, they do not establish causality and therefore cannot demonstrate that these variants are responsible for the patients' clinical phenotypes.

Accordingly, I recommend substantial downtoning of the human genetics claims throughout the manuscript. In particular, statements implying that the identified variants provide a molecular explanation for interferonopathies, define a novel monogenic disorder, or explain the observed patient phenotypes are not sufficiently supported by the available evidence. As causality has not been established, the reported clinical manifestations cannot currently be interpreted as consequences of AZI2 variation. The data support disruption of NAP1 biology by these variants, but do not demonstrate that they are disease-causing in the reported patients.

We thank the reviewer for this careful assessment of the genetic evidence. We agree that the currently available human data do not establish *AZI2* as a definitive disease-causing gene. Indeed, In the previous version of the manuscript, we had already addressed the reviewer's comment by using only the term "*predisposition*". Indeed, the final paragraph of the initially submitted manuscript (now lines 592–596) already clarified this point.

"Finally, since the AZI2 mutations are inherited from an asymptomatic parent, this indicates that additional modifiers, either intrinsic (genetic or epigenetic) or extrinsic (environmental) are probably involved in the pathophysiological mechanisms underlying disease onset. Further studies, pending the availability of patients' material, are needed to explore in detail the consequences of these mutations in patient cells."

However, upon re-reading, we agree with the reviewer that some phrasing remained ambiguous. We have modified the manuscript accordingly to reflect this point more explicitly.

In the abstract (lines 45-48):

The initial text

"*This study reveals an original mode of regulating a signaling pathway by formation of condensates and provides a molecular explanation for certain interferonopathies*"

has been replaced by

"*This study reveals an original mode of regulating a signaling pathway by formation of condensates and provides a potential molecular explanation for immune dysregulation associated with NAP1 variants in certain patients with interferonopathies*"

At the end of the introduction (lines 110-112):

The initial text

"*Our work highlights a novel and original way of regulating the activity of an enzyme by the formation of liquid condensates and provides a pathophysiological mechanism in patients with interferonopathies or lupus.*"

has been replaced by

"*Our work highlights a novel and original way for regulating enzyme activity via liquid condensate formation and identifies a potential pathophysiological mechanism relevant in some patients with interferonopathies or lupus harboring NAP1 variants.*"

In the title of the last section of the results (line 457):

The initial text

"*NAP1 variants are found in patients with immune-related dysregulations*"

has been replaced by

"*Rare NAP1 variants are found in patients with immune-related dysregulations*"

Line 465-466:

The initial text

"*supporting a potential causal role in the pathology*"

has been replaced by

"further supporting their potential contribution to the pathology."

Line 475:

The initial text

"To further support the causal role of these variants,"

has been replaced by

"To further support the contribution of these variants,"

In the discussion (lines 546-548):

The initial text

"The identification of mono-allelic AZI2 mutations in patients with autoimmune or inflammatory disorders, such as SLE and idiopathic interferonopathy, suggested a novel monogenic predisposition to IFN-related diseases."

has been replaced by

"The identification of mono-allelic AZI2 mutations in patients with autoimmune or inflammatory disorders, such as SLE and idiopathic interferonopathy, suggests that AZI2 mutations may confer susceptibility to IFN-related diseases."

In figure 7 legend (lines 1360-1362)

The initial text

"Figure 7| NAP1 variants, associated with immune diseases in patients, have a defect in condensate formation and the downregulation of TBK1-dependent IFN-β pathway
A) NAP1 variants identified in immune diseases.."

has been replaced by

"Figure 7| Patients-derived NAP1 variants have a defect in condensate formation and fail to downregulate TBK1-dependent IFN-β pathway
A) NAP1 variants identified in patients with immune diseases."

We believe this revision accurately reflects the current level of evidence and preserves the value of the study by distinguishing clearly between mechanistic insight and genetic proof of disease causation. The data advance our understanding of how *AZI2* variants may contribute to immune dysregulation, laying the groundwork for future studies to confirm their role in disease.
